# The Role of Circulating Protein and Metabolite Biomarkers in the Development of Pancreatic Ductal Adenocarcinoma (PDAC): A Systematic Review and Meta-analysis

**DOI:** 10.1158/1055-9965.EPI-21-0616

**Published:** 2021-11-22

**Authors:** Swati Kumar, Ralph J. Santos, Andrew J. McGuigan, Urvashi Singh, Peter Johnson, Andrew T. Kunzmann, Richard C. Turkington

**Affiliations:** 1 Patrick G Johnston Centre for Cancer Research, Queen's University Belfast, Belfast, Northern Ireland, United Kingdom.; 2 Centre for Public Health, Queen's University Belfast, Belfast, Northern Ireland, United Kingdom.

## Abstract

**Background:**

Pancreatic ductal adenocarcinoma (PDAC) has a poor prognosis, and this is attributed to it being diagnosed at an advanced stage. Understanding the pathways involved in initial development may improve early detection strategies. This systematic review assessed the association between circulating protein and metabolite biomarkers and PDAC development.

**Methods:**

A literature search until August 2020 in MEDLINE, EMBASE, and Web of Science was performed. Studies were included if they assessed circulating blood, urine, or salivary biomarkers and their association with PDAC risk. Quality was assessed using the Newcastle-Ottawa scale for cohort studies. Random-effects meta-analyses were used to calculate pooled relative risk.

**Results:**

A total of 65 studies were included. Higher levels of glucose were found to be positively associated with risk of developing PDAC [*n* = 4 studies; pooled relative risk (RR): 1.61; 95% CI: 1.16–2.22]. Additionally, an inverse association was seen with pyridoxal 5′-phosphate (PLP) levels (*n* = 4 studies; RR: 0.62; 95% CI: 0.44–0.87). Meta-analyses showed no association between levels of C-peptide, members of the insulin growth factor signaling pathway, C-reactive protein, adiponectin, 25-hydroxyvitamin D, and folate/homocysteine and PDAC risk. Four individual studies also reported a suggestive positive association of branched-chain amino acids with PDAC risk, but due to differences in measures reported, a meta-analysis could not be performed.

**Conclusions:**

Our pooled analysis demonstrates that higher serum glucose levels and lower levels of PLP are associated with risk of PDAC.

**Impact:**

Glucose and PLP levels are associated with PDAC risk. More prospective studies are required to identify biomarkers for early detection.

## Introduction

Pancreatic cancer is considered to be one of the most lethal cancers with a very poor prognosis, and only 5% of patients survive for 10 or more years after diagnosis (1). Pancreatic ductal adenocarcinoma (PDAC) is the most commonly diagnosed pathologic subtype and is estimated to become the second leading cause of cancer-related deaths in the United States by 2030 (2, 3). This is often because PDAC is diagnosed at an advanced stage when surgical resection, the only potentially curative therapy, is not feasible (4, 5). Therefore, early detection is considered to be of utmost importance in order to improve survival rates.

The incidence of PDAC in the general population is low and accounts for only 3% of all new cancer cases diagnosed in the UK (6). This makes it very challenging for the development of early detection strategies and emphasizes the importance of identifying individuals who are at a higher than average risk of developing PDAC (7). Current strategies are limited to screening individuals with a genetic predisposition and involve the use of endoscopic ultrasound (EUS) in addition to other imaging modalities such as MRI and CT. However, there is an urgent need for the identification of less invasive biomarkers that can be used in combination with these approaches (8–11). Various circulating biomarkers present in blood, urine, or saliva are a less invasive alternative when compared with tissue-based markers.

A majority of the studies on circulating biomarkers associated with PDAC biology are case–control studies that have assessed the biomarker very close to diagnosis and therefore do not provide a lot of information on the initial development of the cancer (12–15). This requires prospective studies that evaluate the role of these biomarkers in prediagnostic samples collected in the years preceding diagnosis, in order to help identify high-risk individuals and aid in early detection. Several large-scale cohort studies looking at the association between various biomarkers and PDAC risk have been published in the recent years, and a thorough assessment of these studies will deepen our understanding of the molecular mechanisms of PDAC development. This systematic review, therefore, aims to collate data from all prospective studies on blood-, saliva-, and urine-based biomarkers, their association with PDAC risk and assess the quality of evidence presented by conducting a meta-analysis.

## Materials and Methods

### Search strategy

To assess the association between circulating biomarkers and risk of developing PDAC, Medline (1974–), Embase (1974–), and Web of Science (1970–) were searched systematically for eligible studies in humans using predefined search terms from date of inception to May 10, 2019. Medical Subject Headings and keywords for cancer, biomarkers, sample (blood/urine/saliva), and early detection/risk were used.

An updated search was performed in all three databases on August 20, 2020, to identify any new articles published before beginning the final analysis. The detailed search strategy for each database is included in Supplementary Table S1, and this protocol was registered in the PROSPERO database (CRD42019141149; ref. 16). Additionally, reference lists and manual searches were used to further identify any missed studies.

### Eligibility criteria

Titles and abstracts of the studies identified by the search were screened for eligibility by two independent reviewers (SK reviewed all studies; RS, AM^c^G, AK, US, and PJ reviewed a subset). Any discrepancies were resolved by discussions with a third reviewer and a consensus was reached.

Eligibility criteria for inclusion in the review were as follows: observational and prospective studies assessing the association between prediagnostic nontissue (blood/urine/saliva) based circulating biomarkers and risk of subsequent development of PDAC. Included studies were required to have a follow-up period of at least six months after biomarker assessment and report on both the measure of association, in terms of odds ratios/hazard ratios and their corresponding 95% confidence intervals, or have enough data for these to be calculated. Case–control/retrospective studies, studies where the biomarker was measured at or close to the time of diagnosis (symptoms were already present) and those on biomarkers of cancer mortality were excluded. Participants/population included anyone with a diagnosis of pancreatic cancer or individuals who had no history of cancer at the time of biomarker assessment.

Because the main focus of the review was to look at protein/metabolite markers, studies assessing blood-based genetic markers, miRNA, and infectious agents were not included in the final analysis, and only abstracts were screened for the biomarker studied.

Full texts of the articles were assessed for eligibility, and data extraction was performed by one reviewer (SK). Extracted data from the individual studies included author names, date of publication, study characteristics, participant details, biomarker assessed, sample type, and outcomes of interest. The data extraction was checked by a second reviewer (RS, AM) to ensure accuracy. Study quality was assessed by using the Newcastle-Ottawa scale for cohort studies (17).

### Statistical analysis

If at least three studies assessed the association between a particular circulating protein/metabolite biomarker and PDAC risk, a random-effects meta-analysis was performed. RevMan 5.4 (RRID:SCR_003581; ref. 18) software was used for data synthesis and calculating pooled relative risks and 95% confidence intervals from the eligible studies. A χ^2^ test was used to investigate heterogeneity and *I*^2^ statistic was calculated to report on variation between the study estimates. Heterogeneity was considered high if *I*^2^ statistic was above 75% (19). If individual studies reported results only stratified by sex, these estimates were first pooled together in an initial meta-analysis, and then this pooled estimate was used in the final meta-analysis.

## Results

**Figure 1.**
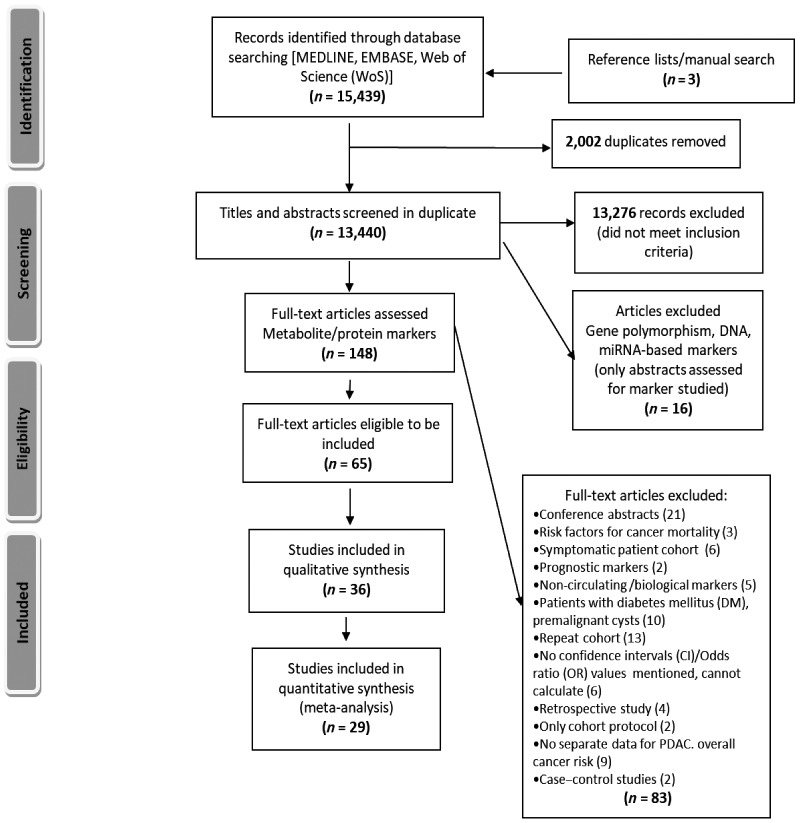
PRISMA flow diagram outlining study selection for the systematic review.

The search identified 15,439 articles from the three databases. After removal of duplicates, the titles and abstracts of 13,437 articles were screened. A total of 145 studies were selected for full-text review, from which 62 studies were deemed eligible for inclusion in the review. An additional three studies were identified by manual searches and review of reference lists, bringing the total number of studies included in the review to 65 (Fig. 1). The characteristics of each of these 65 studies included in the review are summarized in Table 1.

**Table 1.** Summary of characteristics of the studies included in the review.

Reference	Cohort	Country	No. of cases	Age range	Sex	Follow-up period	Biomarkers measured	Method of assessment	Specimen type
Ahn et al. (80)	Alpha-Tocopherol, Beta-Carotene Cancer Prevention Study (ATBC)	Finland	273	50–69 years	Men	Median: 14.9 years	Total and high-density lipoprotein cholesterol		Serum
Arendt et al. (35)	The Health Improvement Network (THIN)	UK	844	18–99 years	Both	Median: 2.8 years	Vitamin B_12_		Plasma
Babic et al. (49)	Health Professionals Follow-Up Study (HPFS), Nurses' Health Study (NHS), Physicians' Health Study (PHS), Women's Health Initiative-Observational Study (WHI-OS), Women's Health Study (WHS)	United States	470	30–84 years	Both	Median: 7.1 years	Leptin		Plasma
Banim et al. (36)	European Prospective Investigation into Cancer and Nutrition (EPIC)-Norfolk	UK	76	40–74 years	Both	17 years max	Vitamin C		Serum
Banim et al. (24)	European Prospective Investigation into Cancer and Nutrition (EPIC)-Norfolk	UK	35	40–74 years	Both	17 years max	Glycosylated hemoglobin (HbA1c)	HPLC	Serum
Bao et al. (102)	HPFS, NHS, PHS, WHI, WHS	United States	470	30–84 years	Both	Median: 7.2 years	CRP, IL6, and TNFαR2		Plasma
Bao et al. (103)	HPFS, NHS, PHS, WHI, WHS	United States	468	30–84 years	Both	Up to 26 years	Adiponectin		Plasma
Chatterjee et al. (57)	The Prostate, Lung, Colorectal and Ovarian Cancer Screening Trial (PLCO)	United States	303	55–70 years	Both	Median: 7 years	Selenium		Serum
Chen et al. (82)	THIN	UK	1,241	60–80 years	Both	Mean: 7.2 years	Cholesterol		Serum
Chuang et al. (41)	EPIC	Multiple	463	25–70 years	Both	Mean: 9.6 years	One-carbon metabolites	Mass spectrometry and microbiological methods	Plasma
Cui et al. (104)	Shanghai WHS, Shanghai Men's Health Study	China	239	40–74 years	Both	Median 5.8 years	Prostaglandin E2 metabolites (PGE-M)	LC-MS/MS	Urine
De Gonzalez et al. (105)	Korean Cancer Prevention Study (KCPS)	Korea	2,194	45+ years	Both	Median: 12 years	Aspartate aminotransferase and alanine aminotransferase		Serum
Douglas et al. (53)	Alpha-Tocopherol, Beta-Carotene (ATBC) Cancer Prevention Study cohort, The Prostate, Lung, Colorectal and Ovarian Cancer Screening Trial (PLCO)	Finland, United States	493	50–74 years	Both	ATBC: Median: 9.4 years	C-reactive protein	ELISA	Serum
						PLCO: Median: 5.4 years			
Douglas et al. (72)	PLCO	United States	187	55–74 years	Both	Median: 5.4 years	IGF-I, IGF-II, IGFBP-3, and IGF-I/IGFBP-3 molar	ELISA	Serum
Gaur et al. (56)	Swedish Apolipoprotein Mortality Risk (AMORIS)	Sweden	197	20 or older	Both	Mean: 10.57 years	Iron, total iron binding capacity (TIBC)		Serum
Grote et al. (47)	EPIC	Multiple	452	35–70 years	Both	Mean: 5.3 years	Adiponectin	Multiplex immunoassay	Serum
Grote et al. (52)	EPIC	Multiple	454	30–76 years	Both	Mean: 5.3 years	Ne-(carboxymethyl)lysine (CML) and the endogenous secreted receptor for AGE (esRAGE)	ELISA	Serum, plasma
Grote et al. (106)	EPIC	Multiple	455	Mean: 58 years	Both	Mean: 5.3 years	C-reactive protein (CRP), IL6, and soluble receptors of tumor necrosis factor-α (sTNFR1, R2)	Immunoassays	Serum
Grote et al. (107)	EPIC	Multiple	466	35–70 years	Both	Mean: 5.3 years	C-peptide	Radioimmunoassay	Serum
Huang et al. (42)	Shanghai Cohort Study Singapore Chinese Health Study	Singapore, China	187	45–74 years	Both	Up to 23 years	Methionine-related metabolites	LC-MS/MS	Serum
Huang et al. (27)	Shanghai Cohort Study Singapore Chinese Health Study	Singapore, China	187	45–74 years	Both	Shanghai: Mean 12.5 years	B_6_ vitamers (pyridoxal 5′-phosphate, pyridoxal, and 4-pyridoxic acid)	LC-MS/MS	Serum
						Singapore: Mean 6.8 years			
Jacobs et al. (108)	ATBC, The Cancer Prevention Study-II (CPS-II), PLCO	Finland, United States	729	Median age: 62 years	Both	23 years maximum	TGF-β1	ELISA	Serum
Jeurnink et al. (37)	EPIC	Multiple	466	Mean: 57.9 years	Both	Mean: 5.25 years	Micronutrients	HPLC/colorimetric assay	Plasma
Jiao et al. (51)	ATBC	Finland	255	50–69 years	Men	Median: 15 years	CML-AGE, sRAGE	ELISA	Serum
Johansen et al. (62)	The Malmö Preventive Project	Sweden	84	37–60 years	Both	Median: 25 years	Forms of trypsinogen	ELISA	Serum
Johansen et al. (66)	Metabolic Syndrome and Cancer Project (Me-Can)	Multiple	862	30–59 years	Both	Mean: Men: 12.8 years; Women: 11.3 years	Glucose, cholesterol, triglycerides	Nonenzymatic/enzymatic method	Serum
Huang et al. (28)	Shanghai Cohort Study Singapore Chinese Health Study	Singapore, China	187	45–74 years	Both	10.7 years	Tryptophan metabolism	LC-MS/MS	Serum
Kabat et al. (81)	Women's Health Initiative	United States	156	50–79 years	Women	Up to 23 years	Total cholesterol, low-density lipoprotein cholesterol (LDL-C), high-density lipoprotein cholesterol (HDL-C), and triglycerides		Serum
Katagiri et al. (44)	Japan Public Health Center	Japan	170	Mean age: 57 years	Both	Median: 10.1 years	Branched-chain amino acids	LC/QqQMS	Plasma
Khalaf et al. (55)	WHI-Observational Study (WHI-OS), HPFS, NHS	United States	396	Mean age: 64 years	Both	Median: 8 years	Salicylurate	LC/MS	Plasma
Kim et al. (109)	HPFS, NHS, PHS, WHI	United States	500	30–84 years	Both	Maximum 26 years	Proinsulin, adiponectin, IL6, and BCAAs		Plasma
Kitahara et al. (46)	National Health Insurance Corporation	Korea	2,575	30 to 95 years	Both	Mean: 12.7 years	Cholesterol		
Laiyemo et al. (63)	ATBC	Finland	227	50–69 years	Men	Mean 10.8 years	Pepsinogen I (SPGI)	Radioimmunoassay	Serum
Leenders et al. (54)	EPIC	Multiple	146	25–70 years	Both	Mean: 8 years	Cotinine	MS	Plasma
Matejcic et al. (39)	EPIC	Multiple	375		Both	Median: 11.7 years	Phospholipid fatty acids	Gas chromatography	Plasma
Mayers et al. (43)	WHI-OS, HPFS, NHS, PHS	United States	453	30–84 years	Both	Median: 8.7 years	Branched-chain amino acids	LC-MS	Plasma
Meinhold et al. (110)	ATBC	Finland	305	50–69 years	Men	Median 16.1 years	HDL-C		Serum
Michaud et al. (25)	WHI-OS, HPFS, NHS, PHS	United States	197	30–84 years	Both	Maximum 20 years	C-peptide, insulin	ELISA	Plasma
Mok et al. (65)	KCPS	Korea		Mean: 41 years	Both	Up to 17 years	Gamma-glutamyltransferase		Serum
Nogueira et al. (26)	ATBC, The Cancer Prevention Study-II (CPS-II), PLCO	Finland, United States	758	50–77 years	Both	Mean: 8.2 years	C-peptide, total and high-molecular-weight adiponectin	ELISA	Serum
Olson et al. (60)	PLCO	United States	283	55–74 years	Both	Median: 7.8 years	Immunoglobulin e	Fluorescent enzyme immunoassay	Serum
Pang et al. (20)	China Kadoorie Biobank (CKB)	China	512	Mean: 51.5 years	Both	8 years	Glucose	SureStep Plus System	Plasma
Piper et al. (32)	PLCO	United States	295	55–74 years	Both	Up to 15.1 years	Vitamin D binding protein, 25(OH)D	Immunoassay	Serum
Rohrmann et al. (73)	EPIC	Multiple	422	30–76 years	Both	Mean: 5.4 years	IGF-I and IGFBP3	Immunoassay	Serum
Schernhammer et al. (34)	WHI-OS, HPFS, NHS, PHS	United States	208	30–84 years	Both	Median: 5.5 years	Plasma folate, vitamin B_6_, vitamin B_12_, and homocysteine		Plasma
Shu et al. (40)	Shanghai WHS, Shanghai Men's Health Study	China	226	40–74 years	Both	Up to 13 years	Lipids and other metabolites	UPLC-QTOFMS, GC-TOFMS	Plasma
Sollie et al. (111)	AMORIS	Sweden	286	20 or older	Both	Mean: 18.3 years	CRP, albumin, haptoglobin, and leukocytes		Serum
Sollie et al. (59)	AMORIS	Sweden	689	20 or older	Both	Mean: 21.3 years	Immunoglobulin G		Serum
Stolzenberg-Solomon et al. (48)	ATBC, CPS-II, PLCO	Finland, United States	731	Median: 71 years	Both	Mean: 8.3 years	Leptin	ELISA	Serum
Stolzenberg-Solomon et al. (112)	PLCO	United States	184	55–74 years	Both	Median: 5.4 years	25-Hydroxyvitamin D		Serum
Stolzenberg-Solomon et al. (22)	ATBC	Finland	169	50–69 years	Men	Median: 13.8 years	Glucose and insulin, insulin resistance (HOMA-IR)	Immunoenzymatic assay	Serum
Stolzenberg-Solomon et al. (33)	ATBC	Finland	126	50–69 years	Men	7–10 years	Homocysteine, vitamin B_12_, folate, PLP, and creatinine		Serum
Stolzenberg-Solomon et al. (75)	ATBC	Finland	93	50–69 years	Men	Up to 12.7 years	IGF-1, IGF-binding protein-3	ELISA	Serum
Stolzenberg-Solomon et al. (38)	ATBC	Finland	306	50–69 years	Men	Median: 16 years	Alpha-tocopherol	HPLC	Serum
Stolzenberg-Solomon et al. (29)	ATBC	Finland	184	50–69 years	Men	Median: 11.8 years	25-Hydroxyvitamin D	RIA	Serum
Stolzenberg-Solomon et al. (45)	ATBC, PLCO	Finland, United States	479	50–79 years	Both	Up to 24 years	Metabolites	LC-MS/MS	Serum
Jee et al. (21)	KCPS	Korea		30–95 years	Both	Up to 10 years	Glucose	Serum	Blood, urine
Sun et al. (61)	Southern Community Cohort Study (SCCS)	United States	73		Both	Median: 4 years	Autoantibodies to Ezrin	ELISA	Plasma
Tsuboya et al. (64)	Ohsaki Cohort Study	Japan	67	40–79 years	Both	Up to 10 years	Gamma-glutamyltransferase	Szasz method	Serum
Weinstein et al. (31)	ATBC	Finland	234	50–69 years	Men	At least 10 years	Vitamin D binding protein	RIA	Serum
White et al. (50)	Women's Health Initiative Study	United States	472	50–79 years	Women	At least 10 years	sRAGE, adipokines	Immunoassay	Serum
Wolpin et al. (23)	WHI-OS, HPFS, NHS, PHS, WHS	United States	449	30–84 years	Both	Up to 25 years	HbA1c, insulin, proinsulin, and proinsulin-to-insulin ratio		Plasma
Wolpin et al. (74)	WHI-OS, HPFS, NHS, PHS	United States	212	30–84 years	Both	At least 8 years	IGF-1, IGF-2, IGF-binding protein-3	ELISA	Plasma
Wolpin et al. (30)	HPFS, NHS, PHS, WHI, WHS	United States	451	Median: 62.5 years	Both	Median: 14.3 years	25-Hydroxyvitamin D	Immunoassay	Plasma
Wulaningsih et al. (58)	AMORIS	Sweden	762	20 or older	Both	Mean: 12.75 years	Inorganic phosphate		Serum

Abbreviations: AMORIS, Swedish Apolipoprotein Mortality Risk; ATBC, Alpha-Tocopherol, Beta-Carotene Cancer Prevention Study; CPS-II, The Cancer Prevention Study-II; EPIC, European Prospective Investigation into Cancer and Nutrition; HPFS, Health Professionals Follow-Up Study; KCPS, Korean Cancer Prevention Study; NHS, Nurses' Health Study; PHS, Physicians' Health Study; PLCO, The Prostate, Lung, Colorectal and Ovarian Cancer Screening Trial; THIN, The Health Improvement Network; WHI, Women's Health Initiative Study; WHI-OS, Women's Health Initiative-Observational Study; WHS, Women's Health Study.

The Newcastle-Ottawa scale was used for assessing quality of the included studies, and 45 of these were considered to be of good quality and 20 were of fair quality (Supplementary Table S2).

### Glucose metabolism–related biomarkers

**Figure 2.**
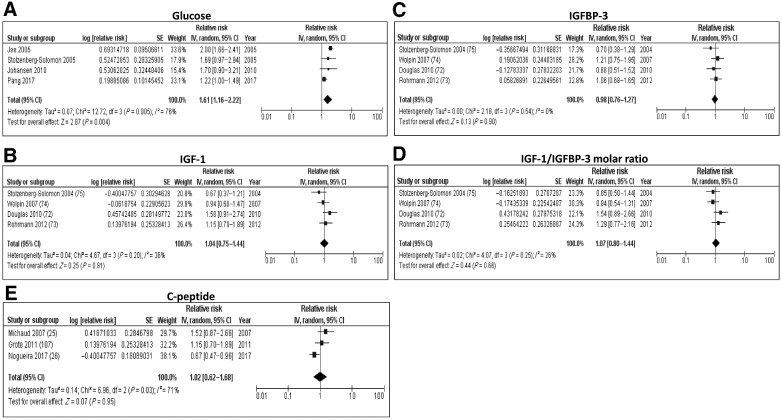
Forest plots from random-effects meta-analysis of the association between glucose metabolism–related biomarkers and PDAC risk. **A,** Glucose; **B**, IGF-1; **C**, IGFBP-3; **D**, IGF-1/IGFBP-3 molar ratio; **E**, C-peptide.

We identified four studies that looked at the association between glucose levels and PDAC risk and the meta-analysis showed a positive association, with increased levels of glucose indicating an enhanced risk of early development of PDAC. However, a high degree of heterogeneity was seen [pooled relative risk (RR): 1.61; 95% CI: 1.16–2.22, *I*^2^ = 76%; Fig. 2A; Supplementary Table S3]. Sensitivity analysis revealed that exclusion of results from Pang and colleagues (ref. 20; pooled RR: 1.95; 95% CI: 1.64–2.31; *I*^2^ = 0%) or Jee and colleagues (ref. 21; pooled RR: 1.30; 95% CI: 1.08–1.55; *I*^2^ = 0%) lowered the heterogeneity observed and the positive association remained. Two studies reported consistent positive association between circulating levels of insulin (22, 23) and HbA1c (23, 24) with PDAC risk, but due to an insufficient number of studies, a meta-analysis could not be performed.

We also identified four studies that looked at circulating levels of various components of the insulin growth factor (IGF) axis and their association with PDAC risk. The meta-analyses showed no significant association between levels of IGF-1 (RR: 1.04; 95% CI: 0.75–1.44, *I*^2^ = 36%; Fig. 2B), IGF-binding protein-3 (IGFBP-3; RR: 0.98; 95% CI: 0.76–1.27, *I*^2^ = 0%; Fig. 2C) and the IGF-1/IGFBP-3 molar ratio (RR: 1.07; 95% CI: 0.80–1.44, *I*^2^ = 26%; Fig. 2D).

Furthermore, three studies looked at the association between circulating C-peptide levels and PDAC risk. The meta-analysis showed no evidence of an association with PDAC risk (RR: 1.02; 95% CI: 0.62–1.68, *I*^2^ = 71%; Fig. 2E) with a certain degree of heterogeneity seen. Michaud and colleagues reported a positive association that was stronger among nonsmokers, and this was seen only in nonfasting blood samples (25). On the other hand, the study by Nogueira and colleagues found an inverse association between plasma C-peptide levels and PDAC risk in current smokers and no association in never or former-smokers (26). These results indicate that the association of C-peptide levels with PDAC risk is somewhat dependent on smoking status, but further investigation into its role in PDAC development is required.

### Nutrition-related markers

**Figure 3.**
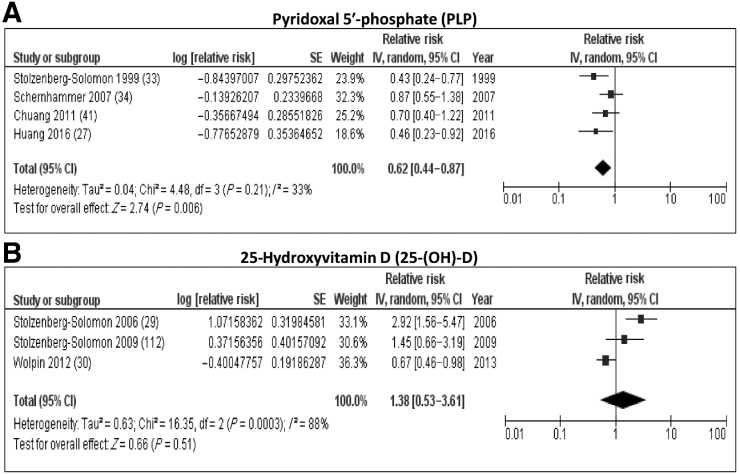
Forest plots from random-effects meta-analysis of the association between nutrition-related biomarkers and PDAC risk. **A,** Pyridoxal 5′-phosphate (PLP) and (**B**) 25-hydroxyvitamin D [25-(OH)-D].

We identified 17 studies that assessed the association between different nutrition-related biomarkers and PDAC risk, and these are listed in Supplementary Table S4. A meta-analysis of four studies found a significant inverse association between levels of circulating pyridoxal 5′-phosphate (PLP), which is the active form of vitamin B6 and risk of PDAC development (RR: 0.62; 95% CI: 0.44–0.87; *I*^2^ = 33%; Fig. 3A). Huang and colleagues also assessed the association between other forms of vitamin B6 vitamers (27) and the role of markers of the kynerurine pathway considered to be functional measures of PLP (28) but a meta-analysis could not be carried out due to insufficient studies (*n* < 3).

We also conducted a meta-analysis of three studies and found that there was no association between levels of 25-hydroxyvitamin D (25-(OH)-D) and risk of developing PDAC (RR: 1.38; 95% CI: 0.53–3.61; *I^2^* = 88%; Fig. 3B) with a high degree of heterogeneity seen. One of the studies (29) included in the meta-analysis reported a significant increased risk of PDAC with increasing 25-(OH)-D levels, whereas another found a decreased risk (30). Additionally, Weinstein and colleagues investigated the association between vitamin D binding protein (DBP), which is the primary carrier of various vitamin D forms and PDAC risk and found an inverse association, which was particularly evident in men with high 25-(OH)-D levels. This observation was accompanied by the reports of higher risk of PDAC in men with an elevated 25(OH)D:DBP molar ratio, which is a proxy for free 25(OH)D (31). Contrasting results were reported by another study that found a positive association between vitamin DBP levels and PDAC risk (32). Two studies reported no association between vitamin B12 levels and PDAC risk (33, 34). Another study found a positive association with PDAC risk, but a meta-analysis was not carried out as the reporting measures between the three studies were different (35).

Vitamin C levels and their association with PDAC risk were investigated by two studies, with one of them reporting an inverse association (36), while the other found no association (37). In addition, we also found two studies that reported inverse associations between a-tocopherol levels and PDAC risk (37, 38)

### Metabolism-related biomarkers

**Figure 4.**
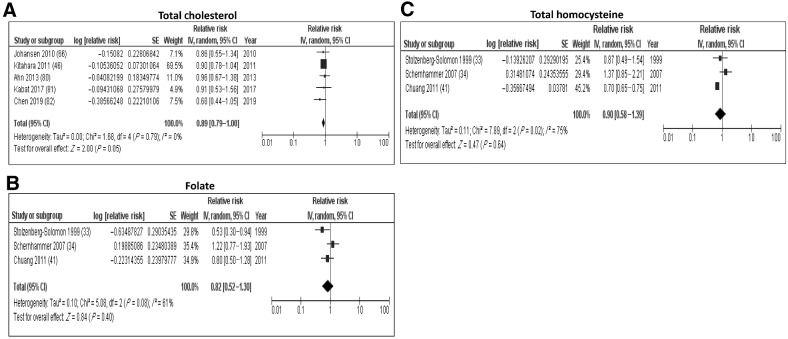
Forest plots from random-effects meta-analysis of the association between metabolism-related biomarkers and PDAC risk. **A,** Total cholesterol; **B**, folate; **C**, total homocysteine.

We identified 17 studies that looked at the association between metabolism-related biomarkers and PDAC risk (Supplementary Table S5). A meta-analysis of five studies found a trend toward an inverse association between levels of total serum cholesterol and risk of PDAC [pooled relative risk (RR): 0.89 95% Confidence Interval (CI): 0.79–1.00, *I*^2^ = 0%); Fig. 4A]. Two of these studies also reported no association between levels of HDL-cholesterol (HDL-C) and triglycerides and PDAC risk. Additionally, Matejcic and colleagues (39) and Shu and colleagues (40) looked at levels of lipid-metabolism–related biomarkers and found inverse associations with PDAC risk for a number of glycerophospholipids and fatty acids.

We also identified four studies that assessed the role of metabolites involved in the one-carbon metabolite pathway in the early development of PDAC. A meta-analysis of three studies found no association between the levels of folate (RR: 0.82; CI: 0.52–1.30; *I*^2^ = 61%; Fig. 4B) and homocysteine (RR: 0.90; CI: 0.58–1.39; *I*^2^ = 75%; Fig. 4C) with PDAC risk, and a high degree of heterogeneity was observed. Interestingly, Chuang and colleagues found a U-shaped dose–response relationship between plasma levels of folate and risk, with a significant inverse association observed for the fourth versus first quintile (OR = 0.5; 95% CI = 0.3–0.8), while there was no clear association for the fifth versus first quintile (OR = 0.8; 95% CI = 0.5–1.4; ref. 41). Two studies also assessed the association between methionine levels and PDAC risk but reported conflicting results with one study identifying a positive association in men (41), whereas a significant inverse association with PDAC risk was observed in the other study (42).

We also identified four studies that reported on the association between circulating branched-chain amino acids and PDAC risk. A prospective study of four large cohorts found that increased levels of the BCAAs leucine, isoleucine, and valine were associated with at least a 2-fold increased risk of developing PDAC. The levels of these markers were highly correlated and therefore showed a similar positive association for the sum total of the BCAAs as well (43). These interesting findings were supported by another study based in Japan, which also found that higher levels of the BCAAs were associated with an increased PDAC risk (44). The study by Shu and colleagues, which reported the association between several glycerophospholipids with PDAC risk, also identified BCAAs in their sample cohort, and this indicated a positive association that was stronger in subjects whose cancer was diagnosed early, but was not statistically significant (40). A similar positive association for the BCAAs was also reported in the large metabolomics study by Stolzenberg-Solomon and colleagues, but this did not pass their multiple comparison significance threshold (45). These are promising findings that provide evidence for the probable role of BCAAs in the early development of PDAC and should be investigated further. However, a meta-analysis could not be performed as the studies reported results/measures stratified differently, with Shu and colleagues (40) only reporting odds ratios stratified by follow-up time for the individual BCAAs, while Kitahara and colleagues (46) reported odds ratio stratified by follow-up time for the sum total of the BCAAs.

### Inflammation-related markers

**Figure 5.**
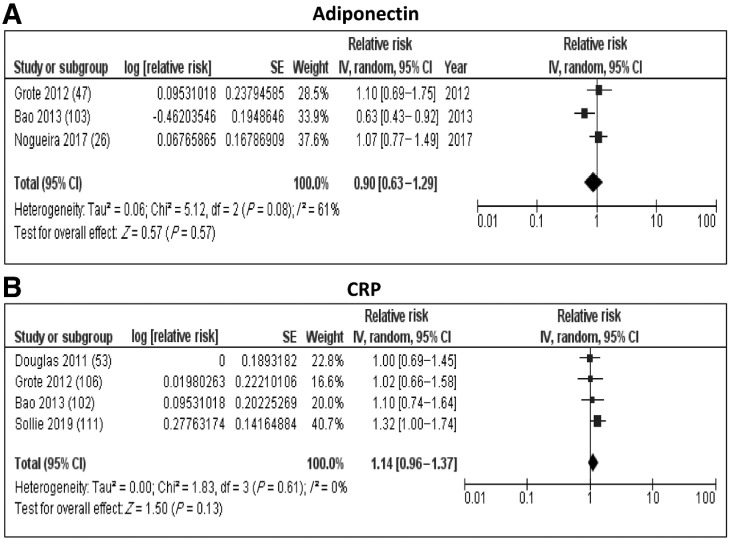
Forest plots from random-effects meta-analysis of the association between inflammation-related biomarkers and PDAC risk. **A,** Adiponectin and (**B**) C-reactive protein.

We identified 13 studies that looked at the association between levels of inflammation-related markers and the risk of developing PDAC (Supplementary Table S6). A meta-analysis of three studies looking at circulating adiponectin levels found no association with PDAC risk (RR: 0.90; 95% CI: 0.63–1.29; *I*^2^ = 61%; Fig. 5A) and also showed a certain degree of heterogeneity. Interestingly, like C-peptide levels, two studies reported an inverse association between circulating levels of adiponectin and PDAC risk, which was specific to never smokers (26, 47). We also identified two studies that looked at the association between another adipokine, leptin and PDAC risk and one of them reported no clear overall association (48), whereas the second study by Babic and colleagues found higher levels of leptin, indicating an increased risk in men but not in women (49). In addition, markers involved in the receptor for the advanced glycation end products (RAGE) pathway were assessed with baseline soluble RAGE (sRAGE) levels found to be inversely associated with PDAC risk in two studies (50, 51), and conflicting results were reported on the association between Nε-carboxymethyl-lysine (CML)-AGE, which is one of the best characterized AGEs (51, 52).

We also identified four studies that looked at the association between the chronic inflammatory marker C-reactive protein (CRP) and PDAC risk. A meta-analysis found no association between the levels of this marker and pancreatic cancer risk (RR: 1.17; 95% CI: 0.96–1.42; *I*^2^ = 0%; Fig. 5B). However, in two nested case–control studies in the ATBC and PLCO cohorts, an inverse association was reported in younger participants (<66 years), which was not seen in the older group (66 or older; ref. 53).

### Miscellaneous markers

We also identified 11 studies that assessed the association between biomarkers involved in tobacco metabolism (45, 54, 55), micronutrients (56–58), immunoglobulins (59–61), enzymes such as trypsinogen (62), pepsinogen (63), and gamma-glutamyl transferase (64, 65). These results are summarized in Supplementary Table S7.

## Discussion

The aim of this systematic review was to assess the association of various circulating biomarkers with PDAC risk, in order to understand their role in the early development of this cancer. We identified 65 eligible articles, and meta-analysis showed a positive association between glucose levels and PDAC risk (*n* = 4 studies). Additionally, an inverse association was found between levels of cholesterol (*n* = 5 studies) and PLP (*n* = 4 studies).

The positive association seen between levels of glucose and PDAC in the meta-analysis of four studies (20–22, 66) showed a large degree of heterogeneity. This association remained consistent despite geographical differences among the four studies, with two studies being conducted in Europe and the other two in Asia. However, sensitivity analysis showed that heterogeneity was lowered after exclusion of the results from Pang and colleagues (20) or Jee and colleagues (21), and the positive association remained. Three of these studies used fasting serum samples for the measurement of glucose levels and also excluded cases diagnosed early on in the follow-up period (lag ranged from within 1–5 years of follow-up), which could indicate that the associations seen between increasing glucose levels and PDAC risk are not a consequence of PDAC development or an early marker of disease (21, 22, 66). Pang and colleagues (20), on the other hand, measured glucose levels as random blood glucose in nonfasting samples. For the studies that used fasting blood samples, detailed information on fasting time was not provided, and this could influence the results seen in the meta-analysis as metabolite levels can be significantly affected by sampling conditions. Additionally, two of the studies included in the meta-analysis (22, 66) included adjustment for BMI, whereas both Pang and colleagues and Jee and colleagues did not, which could further explain the heterogeneity seen. These findings are in line with a systematic review of prospective observational studies published on the association between fasting blood glucose and risk of pancreatic cancer. This review included studies on PDAC mortality and those which estimated glucose levels from Hb1Ac values, which were not done in our study. They reported a 14% increase in PDAC risk with increasing glucose levels in their meta-analysis of nine studies (67). These results provide strong evidence of an association between glucose levels and PDAC risk; however, further understanding on the nature of this relationship is necessary to strengthen our knowledge on the molecular development of PDAC. Additionally, a number of studies identified in our review reported positive associations between levels of insulin, C-peptide as well as proinsulin and HOMA scores with PDAC risk (22, 23, 25, 26). As impaired β-cell function and insulin resistance have been reported to play a role in the glucose intolerance seen in pancreatic cancer, these findings suggest a close interaction between these pathways could play an important role in early development of PDAC (68, 69).

The IGF axis is another pathway that is closely related to insulin resistance. Insulin has been reported to increase the levels of biologically active IGF-1 and can also alter concentrations of its binding proteins (IGFBP; refs. 70, 71). In our review, results from four studies were consistent, and the meta-analyses showed no significant association between levels of IGF-1, IGFBP-3, and the IGF-1/IGFBP-3 molar ratio (72–75). Similar observations were made in a systematic review and meta-analysis on the association between the IGF-axis and PDAC risk; however, this included retrospective case–control studies as well (76). Although these findings suggest that the IGF axis plays a minimal role in the initial development of PDAC, other studies on genetic variants of different members of this axis have reported significant associations with PDAC risk as well as clinical outcomes, and therefore more studies are needed to make a proper conclusion on the role of this axis in PDAC development (77–79).

We also identified several studies that looked at different metabolism-related markers. A meta-analysis of five studies (46, 66, 80–82) showed a trend toward an inverse association between cholesterol levels and PDAC risk. However, only two of these studies included results that were independent of statin use, which could affect the overall association and could act as a confounding factor (81, 82). Moreover, four studies reported on overall cancer incidence, and only one of these studies was specific to pancreatic cancer and included follow-up data that suggested that the inverse association was attenuated as follow-up time increased. These results are in accordance with other cohort studies on cancer incidence, in which the association between cholesterol levels and cancer risk decreased as follow-up time increased or when cases diagnosed in the first few years after follow-up were excluded and could indicate that this association was likely a consequence of preclinical disease (80, 83, 84). These findings should however be interpreted with caution as other studies have also reported persistent associations with longer follow-up times and therefore more research is required on specific cancer sites in order to draw definitive conclusions (85–87). We also identified four studies that reported on the association between circulating branched-chain amino acids and PDAC risk. BCAAs have been reported to be elevated in individuals who are obese and with insulin resistance and also said to be associated with future development of diabetes (88–90). Because these are all considered to be risk factors for PDAC, BCAAs could play an important role in the initial development of PDAC and should be researched further.

Finally, our review explored the role of various biomarkers involved in the one-carbon metabolism pathway. No association was found in our study for folate or total homocysteine, but we found a significant inverse association between circulating levels of PLP (active form of vitamin B6) and PDAC risk in a meta-analysis of four studies (27, 33, 34, 41). All four studies reported no change in the inverse association seen on excluding cases diagnosed within two to four years of follow-up time, minimizing the bias contributed by reverse causality. Additionally, all studies except one (33) were able to adjust for risk factors of PDAC such as BMI and history of diabetes. However, only two studies (33, 34) had data on multivitamin supplement use, which could be a potential confounding factor. Dietary or circulating nutrients such as folate, methionine, vitamin B12, and B6 are considered to be potential risk factors for cancer and may have protective functions through their role in facilitating DNA methylation, nucleotide synthesis, DNA repair, and replication. Both vitamin B6 and B12 act as cofactors for various important reactions in this pathway (91, 92). Vitamin B6 has been reported to help protect against DNA damage and is also a cofactor involved in the production of the antioxidant glutathione. It may also serve as a scavenger of reactive oxygen species in addition to its role as a cofactor. It has also been shown that deficiency in PLP leads to accumulation of AGEs that increase genomic instability by elevating oxidative stress (93–95). Interestingly, levels of receptors for AGEs (RAGE) have been reported to be inversely associated with PDAC risk as identified in our review (50–52). These results are in line with two other systematic reviews assessing the association between dietary PLP intake (96, 97) and circulating PLP (97) with PDAC risk, and both report a significant inverse association suggestive of a protective role in PDAC development. The review on blood PLP levels also included one case–control study which did not meet our inclusion criteria (97). This protective function of vitamin B6 has also been reported for other cancers and further emphasizes the importance of studying the underlying role of these metabolites and their associated pathways in the early development of PDAC (98–101).

The main strength of this review is that, to the best of our knowledge, it is the first comprehensive systematic review and meta-analysis conducted on the association of circulating biomarkers as a whole and PDAC risk. The quality and risk of bias was assessed using the established tool NOS (17) and several studies earned a good score (<7). Additionally, the funnel plot of the included studies was symmetrical, indicating that there are fewer chances of introduction of publication bias (Supplementary Fig. S1). We also included only prospective cohort studies with a follow-up period of at least six months in order to ensure that the biomarkers are assessed not very close to diagnosis of PDAC so as to gain a better understanding on the biological pathways involved in early PDAC development. Despite the large number of studies identified in our review, very few studies have looked at the same biomarker in order to draw out definite conclusions. This is especially true in terms of the metabolite biomarkers studied and our review has helped discover this limitation, and therefore more studies focused on these biomarkers should be carried out. In terms of limitations, the studies included in the review measured the biomarker only once at baseline in prediagnostic samples, and longitudinal assessment of the biomarker in the same individual might provide greater insight into its role in cancer development. Additionally, a few studies assessing the same biomarker which were used in the meta-analyses had reported varying cutoff values for biomarker concentrations in terms of quartiles, tertiles, etc., and this could also potentially influence the associations observed.

In summary, our findings strengthened the evidence on the role of increasing glucose levels with PDAC development and also discovered an inverse association with PLP levels. We also identified a possible role of BCAAs and trend toward the role of low levels of cholesterol in the early development of PDAC. However, further research is required in order to draw definitive conclusions on the nature of these relationships and deepen our understanding of the role of these pathways in governing PDAC biology.

## Authors' Disclosures

S. Kumar reports grants and personal fees from European Union Horizon 2020 Marie Sklodowska-Curie Actions COFUND Doctoral training program during the conduct of the study. A.J. McGuigan reports grants from Royal College of Surgeons and Ulster Society of Gastroenterology during the conduct of the study. R.C. Turkington reports personal fees from Almac Diagnostics outside the submitted work. No disclosures were reported by the other authors.

## Supplementary Material

Supplementary Data

Supplementary Data

Supplementary Data

Supplementary Data

Supplementary Data

Supplementary Data

Supplementary Data

Supplementary Data
