# Supplementary material for: The Role of Circulating Protein and Metabolite Biomarkers in the Development of Pancreatic Ductal Adenocarcinoma (PDAC): A Systematic Review and Meta-analysis
Source: Cancer Epidemiol Biomarkers Prev. 2021 Nov 22;31(5):1090–102. doi: 10.1158/1055-9965.EPI-21-0616 (PMC9377754; doi:10.1158/1055-9965.EPI-21-0616)
Supplement: Supplementary Data [file epi-21-0616_supp1.docx]

| **Medline** |
| --- |
| **Pancreas terms** |
| 1. exp pancreatic neoplasms/ |
| 2. pancreatic*.mp. |
| 3. ''pancreatic ductal''*.mp. |
| 4. 1 or 2 or 3 |
| **Cancer terms** |
| 5. carcinoma*.mp |
| 6. neoplasm/ |
| 7. cancer.mp |
| 8. neoplas*.mp |
| 9. carcinoma/ |
| 10. adenocarcinoma*.mp. |
| 11. malignan*.mp |
| 12. tumo?r*.mp |
| 13. 5 or 6 or 7 or 8 or 9 or 10 or 11 or 12 |
| **Early detection terms** |
| 14. "Early Detection of Cancer"/ |
| 15. exp diagnosis/ |
| 16. risk/ |
| 17. detect*.mp |
| 18. predict*.mp |
| 19. exp sensitivity/ and specificity.mp. |
| 20. sensitivity*.mp. |
| 21. specificity*.mp. |
| 22. roc curve/ |
| 23. ''Receiver operating characteristic''.mp |
| 24. ''area under the curve''.mp. |
| 25. exp Area Under Curve/ |
| 26. AUC.mp. |
| 27. AUROC.mp. |
| 28. c statistic.mp |
| 29. stratif*.mp |
| 30. discriminat*.mp |
| 31. reclassif*.mp |
| 32. 14 or 15 or 16 or 17 or 18 or 19 or 20 or 21 or 22 or 23 or 24 or 25 or 26 or 27 or 28 or 29 or 30 or 31 |
| **Marker terms** |
| 33. molecular marker.mp |
| 34. biological marker*.mp. |
| 35. biomarker*.mp. |
| 36. exp biological marker/ |
| 37. level*.mp |
| 38. concentration*.mp. |
| **Sample terms** |
| 39. serum.mp |
| 40. plasma.mp |
| 41. exp blood/ |
| 42. serol*.mp |
| 33. saliv* |
| 44. urin*.mp |
| 45. 33 or 34 or 35 or 36 or 37 or 38 |
| 46. 39 or 40 or 41 or 42 or 43 or 44 |
| 47. 4 and 13 and 32 and 45 and 46 |
| 48. Limit 47 to humans |

**a)**

| **Embase** |
| --- |
| **Pancreas terms** |
| 1. exp pancreatic neoplasms/ |
| 2. pancreatic*.mp. |
| 3. ''pancreatic ductal''*.mp. |
| **4. 1 or 2 or 3** |
| **Cancer terms** |
| 5. exp carcinoma/ |
| 6. exp neoplasm/ |
| 7. cancer.mp |
| 8. neoplas*.mp |
| 9. carcinoma/ |
| 10. adenocarcinoma*.mp. |
| 11. malignan*.mp |
| 12. tumo?r*.mp |
| **13. 5 or 6 or 7 or 8 or 9 or 10 or 11 or 12** |
| **Early detection terms** |
| 14. early cancer diagnosis/ |
| 15. exp diagnosis/ |
| 16. risk/ |
| 17. detetct*.mp |
| 18. predict*.mp |
| 20. sensitivity*.mp. |
| 21. specificity*.mp. |
| 22. roc curve/ |
| 23. ''Receiver operating characteristic''.mp |
| 24. ''area under the curve''.mp. |
| 25. exp Area Under Curve/ |
| 26. AUC.mp. |
| 27. AUROC.mp. |
| 28. c statistic.mp |
| 29. stratif*.mp |
| 30. discriminat*.mp |
| 31. reclassif*.mp |
| **32. 14 or 15 or 16 or 17 or 18 or 19 or 20 or 21 or 22 or 23 or 24 or 25 or 26 or 27 or 28 or 29 or 30 or 31** |
| **Marker terms** |
| 33. molecular marker.mp |
| 34. biological marker*.mp. |
| 35. biomarker*.mp. |
| 36. exp biological marker/ |
| 37. level.mp. |
| 38. concentration*.mp. |
| **Sample terms** |
| 39. exp serum/ |
| 40. plasma/ |
| 41. exp blood/ |
| 42. serol*.mp |
| 43.saliv* |
| 44. urin*.mp |
| **45. 33 or 34 or 35 or 36 or 37 or 38** |
| **46. 39 or 40 or 41 or 42 or 43 or 44** |
| **47. 4 and 13 and 32 and 45 and 46** |
| **48. Limit 47 to humans** |

**b)**

| **Web of Science** |
| --- |
| **Pancreas terms** |
| 1. TS=(pancrea* OR pancreatic* OR pancreatic ductal*)) *AND*DOCUMENT TYPES: (Article) |
|  |
| **Cancer terms** |
| 2. TS=(cancer OR carcinoma* OR neoplas* OR malignan* OR tumo$r* OR adenocarcinoma*)) AND **DOCUMENT TYPES**: (Article) |
|  |
| **Sample terms** |
| 3. TS=(serum OR plasma OR blood OR serol* OR saliv* OR urin*)) *AND*DOCUMENT TYPES: (Article) |
|  |
| **Early detection terms** |
| (TS=(“early detection” OR diagnos* OR detect* OR risk OR predict OR sensitivity OR specificity OR “ROC curve” OR “receiver operating characteristic curve” OR “area under the curve” OR AUC OR “c statistic” OR stratif* OR discriminat* OR reclassif*OR level OR levels)) AND DOCUMENT TYPES: (Article) |
| **Marker terms** |
| 5. TS= (biomarker* or marker* of ‘biological marker*’)) *AND***DOCUMENT TYPES**: (Article) |
| **6. #5 AND #4 AND #3 AND #2 AND #1** |

**c)**

**Supplementary Table 1: Database search strategies for a) Medline b) Embase and c) Web of Science for identifying relevant articles**
