# Supplementary material for: The Role of Circulating Protein and Metabolite Biomarkers in the Development of Pancreatic Ductal Adenocarcinoma (PDAC): A Systematic Review and Meta-analysis
Source: Cancer Epidemiol Biomarkers Prev. 2021 Nov 22;31(5):1090–102. doi: 10.1158/1055-9965.EPI-21-0616 (PMC9377754; doi:10.1158/1055-9965.EPI-21-0616)
Supplement: Supplementary Data [file epi-21-0616_supp2.docx]

| Author (Year) | Selection |  |  |  | Comparability  ^b^ | Outcome |  |  | Total  ^d^ |
| --- | --- | --- | --- | --- | --- | --- | --- | --- | --- |
|  | **Representative** | **Selection of**  **non‐cases** | **Exposure**  **ascertainment** | **Outcome not  present ^a^** |  | **Assessment of outcome  ^c^** | **Follow‐up  length** | **Loss to  follow‐up** |  |
| Ahn 2009 | 0 | 1 | 1 | 0 | 2 | 0 | 1 | 1 | 6 |
| Arendt 2019 | 0 | 1 | 1 | 0 | 2 | 0 | 1 | 1 | 6 |
| Babic 2016 | 0 | 1 | 1 | 1 | 2 | 1 | 1 | 1 | 8 |
| Banim 2013 | 1 | 1 | 1 | 1 | 2 | 0 | 1 | 1 | 8 |
| Banim 2018 | 1 | 1 | 1 | 1 | 2 | 0 | 1 | 1 | 8 |
| Bao 2012 | 0 | 1 | 1 | 1 | 2 | 1 | 1 | 1 | 8 |
| Bao 2013 | 0 | 1 | 1 | 1 | 2 | 1 | 1 | 1 | 8 |
| Chatterjee 2019 | 1 | 1 | 1 | 0 | 2 | 1 | 1 | 1 | 8 |
| Chen 2018 | 1 | 1 | 1 | 0 | 2 | 0 | 1 | 1 | 7 |
| Chuang 2011 | 1 | 1 | 1 | 0 | 2 | 1 | 1 | 1 | 8 |
| Cui 2017 | 1 | 1 | 1 | 0 | 2 | 1 | 1 | 1 | 8 |
| Douglas 2010 | 1 | 1 | 1 | 1 | 2 | 1 | 1 | 1 | 9 |
| Douglas 2011 | 0 | 1 | 1 | 0 | 2 | 1 | 1 | 1 | 7 |
| Gaur 2013 | 1 | 1 | 1 | 1 | 1 | 0 | 1 | 1 | 7 |
| Gonzalez 2008 | 0 | 1 | 1 | 1 | 2 | 1 | 1 | 1 | 8 |
| Grote 2011 | 1 | 1 | 1 | 0 | 2 | 0 | 1 | 1 | 7 |
| Grote 2012 (Ref 47) | 1 | 1 | 1 | 0 | 2 | 0 | 1 | 1 | 7 |
| Grote 2012 (Ref 52) | 1 | 1 | 1 | 0 | 2 | 0 | 1 | 1 | 7 |
| Grote 2012 (Ref 106­) | 1 | 1 | 1 | 0 | 2 | 0 | 1 | 1 | 7 |
| Huang 2016 | 1 | 1 | 1 | 0 | 2 | 1 | 1 | 1 | 8 |
| Huang 2018 | 1 | 1 | 1 | 0 | 2 | 1 | 1 | 1 | 8 |
| Huang 2020 | 1 | 1 | 1 | 0 | 2 | 1 | 1 | 1 | 8 |
| Jacobs 2014 | 0 | 1 | 1 | 0 | 2 | 1 | 1 | 1 | 7 |
| Jee 2005 | 0 | 1 | 1 | 0 | 2 | 0 | 1 | 1 | 6 |
| Jeurnink 2015 | 1 | 1 | 1 | 0 | 2 | 1 | 1 | 1 | 8 |
| Jiao 2011 | 0 | 1 | 1 | 1 | 2 | 1 | 1 | 1 | 8 |
| Johansen 2010 (Ref 62) | 1 | 1 | 1 | 1 | 2 | 0 | 1 | 1 | 8 |
| Johansen 2010 (Ref 66) | 1 | 1 | 1 | 1 | 2 | 0 | 1 | 1 | 8 |
| Kabat 2018 | 1 | 1 | 1 | 0 | 2 | 0 | 1 | 1 | 7 |
| Katagiri 2018 | 1 | 1 | 1 | 0 | 2 | 0 | 1 | 1 | 7 |
| Khalaf 2018 | 0 | 1 | 1 | 1 | 2 | 1 | 1 | 1 | 8 |
| Kim 2020 | 0 | 1 | 1 | 0 | 2 | 1 | 1 | 1 | 7 |
| Kitahara 2011 | 0 | 1 | 1 | 0 | 2 | 0 | 1 | 1 | 6 |
| Laiyemo 2009 | 0 | 1 | 1 | 1 | 2 | 1 | 1 | 1 | 8 |
| Leenders 2012 | 1 | 1 | 1 | 1 | 1 | 0 | 1 | 1 | 7 |
| Matejcic 2018 | 1 | 1 | 1 | 0 | 2 | 1 | 1 | 1 | 8 |
| Mayers 2015 | 0 | 1 | 1 | 1 | 2 | 1 | 1 | 1 | 8 |
| Meinhold 2009 | 0 | 1 | 1 | 1 | 2 | 0 | 1 | 1 | 7 |
| Michaud 2007 | 0 | 1 | 1 | 1 | 2 | 1 | 1 | 1 | 8 |
| Mok 2015 | 0 | 1 | 1 | 0 | 2 | 0 | 1 | 1 | 6 |
| Nogueira 2017 | 0 | 1 | 1 | 0 | 2 | 1 | 1 | 1 | 7 |
| Olsen 2014 | 1 | 1 | 1 | 0 | 2 | 1 | 1 | 1 | 8 |
| Pang 2017 | 1 | 1 | 1 | 0 | 2 | 1 | 1 | 1 | 8 |
| Piper 2015 | 1 | 1 | 1 | 1 | 2 | 0 | 1 | 1 | 8 |
| Rohrmann 2012 | 1 | 1 | 1 | 0 | 2 | 1 | 1 | 1 | 8 |
| Schernhammer 2007 | 0 | 1 | 1 | 1 | 2 | 1 | 1 | 1 | 8 |
| Shu 2018 | 1 | 1 | 1 | 0 | 2 | 1 | 1 | 1 | 8 |
| Sollie 2019 | 1 | 1 | 1 | 0 | 1 | 0 | 1 | 1 | 6 |
| Sollie 2020 | 1 | 1 | 1 | 0 | 1 | 0 | 1 | 1 | 6 |
| Stolzenberg-Solomon 1999 | 0 | 1 | 1 | 0 | 1 | 1 | 1 | 1 | 6 |
| Stolzenberg-Solomon 2004 | 0 | 1 | 1 | 1 | 2 | 1 | 1 | 1 | 8 |
| Stolzenberg-Solomon 2005 | 0 | 1 | 1 | 1 | 2 | 1 | 1 | 1 | 8 |
| Stolzenberg-Solomon 2006 | 0 | 1 | 1 | 0 | 2 | 1 | 1 | 1 | 7 |
| Stolzenberg-Solomon 2009 | 0 | 1 | 1 | 0 | 2 | 1 | 1 | 1 | 7 |
| Stolzenberg-Solomon 2010 | 0 | 1 | 1 | 0 | 2 | 0 | 1 | 1 | 6 |
| Stolzenberg-Solomon 2015 | 0 | 1 | 1 | 0 | 2 | 1 | 1 | 1 | 7 |
| Stolzenberg-Solomon 2020 | 0 | 1 | 1 | 0 | 1 | 1 | 1 | 1 | 6 |
| Sun 2016 | 1 | 1 | 1 | 0 | 1 | 1 | 1 | 1 | 7 |
| Tsuboya 2012 | 0 | 1 | 1 | 0 | 2 | 0 | 1 | 1 | 6 |
| Weinstein 2012 | 0 | 1 | 1 | 0 | 2 | 1 | 1 | 1 | 7 |
| White 2018 | 1 | 1 | 1 | 1 | 2 | 1 | 1 | 1 | 9 |
| Wolpin 2007 | 0 | 1 | 1 | 0 | 2 | 1 | 1 | 1 | 7 |
| Wolpin 2012 | 0 | 1 | 1 | 0 | 2 | 1 | 1 | 1 | 7 |
| Wolpin 2013 | 0 | 1 | 1 | 0 | 2 | 1 | 1 | 1 | 7 |
| Wulaningsih 2013 | 1 | 1 | 1 | 0 | 1 | 0 | 1 | 1 | 6 |

**Supplementary Table 2: Newcastle-Ottawa scale for quality assessment**

a: 1 point if cases diagnosed within 1 or more years of follow-up excluded

b: 1 point for age/sex adjustment, 1 point for smoking/BMI

c: 1 point if blinded to case-control status

d: Good quality: 3 or 4 stars in selection domain and 1 or 2 stars in comparability domain and 2 or 3 stars in outcome/exposure domain; fair quality: 2 stars in selection domain and 1 or 2 stars in comparability domain and 2 or 3 stars in outcome/exposure domain; poor quality: 0 or 1 star in selection domain or 0 stars in comparability domain or 0 or 1 stars in outcome/exposure domain.
