# Supplementary material for: The Role of Circulating Protein and Metabolite Biomarkers in the Development of Pancreatic Ductal Adenocarcinoma (PDAC): A Systematic Review and Meta-analysis
Source: Cancer Epidemiol Biomarkers Prev. 2021 Nov 22;31(5):1090–102. doi: 10.1158/1055-9965.EPI-21-0616 (PMC9377754; doi:10.1158/1055-9965.EPI-21-0616)
Supplement: Supplementary Data [file epi-21-0616_supp3.docx]

|  |  | Total cases n | Categorical | | | | | | Continuous | | Adjusted for/stratified by: | | | |  | | | |
| --- | --- | --- | --- | --- | --- | --- | --- | --- | --- | --- | --- | --- | --- | --- | --- | --- | --- | --- |
| Biomarkers |  |  | **Categories** | **High** | **Reference** | **Units** | **RR/HR/OR (95% CI)** | **RR/HR (95% CI)** | | **Age** | | **Sex** | **BMI/WHR** | **Smoking** | | **Alcohol** | **Diabetes** |  |
| HbA1c | Banim, PJR et al.  Wolpin BM et al. | 35  449 | Quintiles  Quintiles | 40 < 151  5.50 | 7 < 30  4.77 | mmol/mol  % | 6.32 (1.38-28.89)  1.79 (1.17 to 2.72) | 1.52, (1.15- 2.03) | | Y  Y | | Y  Y | Y | Y  Y | |  | Y |  |
| Insulin like growth factor family:  Insulin like growth factor (IGF) 1  IGF 2  IGFBP-3  IFG1/IGFBP-3 | Douglas, JB et al.  Rohrmann S et al.  Stolzenberg-Solomon R.Z et al  Wolpin BM et al.  Douglas, JB et al.  Wolpin BM et al.  Douglas, JB et al.  Rohrmann S et al.  Stolzenberg-Solomon R.Z et al  Wolpin BM et al.  Douglas, JB et al.  Rohrmann S et al.  Stolzenberg-Solomon R.Z et al  Wolpin BM et al. | 187  422  93  212  187  212  187  422  93  212  187  422  93  212 | Quartiles  Quartiles  Tertiles  Quartiles  Quartiles  Quartiles  Quartiles  Quartiles  Tertiles  Quartiles  Quartiles  Quartiles  Tertiles  Quartiles | M: >237.8  F:>186.8  M: 227 – 437  F: 221 – 433  >159.1  242.1  M: >1831.8  F:1999.7  1335.0  M: >4181.2  F:>4481.2  M: 5092 – 9367  F: 5342 –11128  >2625  5290.2  M:>0.23  F:>0.16  M: 0.22-0.43  F: 0.22 – 0.44  >0.24  0.19 | M: <151.4  F:<106.4  M: 33 – 138  F: 40 – 128  <121.8  97.1  M:<1339.3  F:<1452.5  802.8  M:<2977.6  F:<3236.8  M: 1625 – 3800  F: 1698 – 4085  <2073  3345.3  M:≤0.17  F:≤0.17  M: 0.05 – 0.15  F: 0.05 – 0.12  <0.20  0.09 | ng/Ml  ng ml^_1^  ng/Ml  ng ml^_1^  ng/mL  ng ml^_1^  ng/mL  ng ml^_1^  ng/mL  ng ml^_1^  ng/mL  Ratio  Ratio  Ratio | 1.58 (0.91–2.76)  1.15 (0.70 – 1.88)  0.67 (0.37– 1.21)  0.94 (0.60 –1.48)  0.86 (0.49–1.50)  0.96 (0.61 –1.52)  0.88 (0.51–1.51)  1.06 (0.68 – 1.65)  0.70 (0.38– 1.27)  1.21 (0.75 –1.92)  1.54 (0.89–2.66)  1.29 (0.77 – 2.16)  0.85 (0.50– 1.46)  0.84 (0.54 –1.31) | - 1. (0.98 – 1.04)  1. (0.99 – 1.01)   1.02 (0.99 – 1.05) | | Y  Y  Y  Y  Y  Y  Y  Y  Y  Y  Y  Y  Y  Y | | Y  Y  Y  Y  Y  Y  Y | Y  Y  Y  Y  Y  Y  Y | Y  Y  Y  Y  Y  Y  Y  Y  Y  Y  Y  Y  Y  Y | |  | Y  Y  Y  Y |  |
| Glucose | Johansen, Dorthe et al.  Stolzenberg-Solomon R.Z et al.  Pang Y. et al  Jee et al. | 862  169  512 | Quintiles  Quartiles | M:6.9  F:7.1  >107  ≥7.0  <90 | M:4.2  F:4.1  <93  <5.5  ≥140 | mmol/L  mmol/L  mg/dL  mmol/L  mg/dL | 1.24(0.95-1.61)  2.39(1.61-3.54)  1.69 (0.97-2.94)  1.22 (1.00, 1.50)  M: 2.09 (1.70-2.58)  F: 1.67 (1.09-2.56) | 1.12 (1.04, 1.21) | | Y  Y  Y  Y  Y | |  | Y  Y  Y | Y  Y  Y  Y  Y | | Y  Y |  |  |
| C peptide | Nogueira L et al.  Michaud DS et al  Grote V.A et al. | 758  197  466 | Quintiles  Quartiles  Quartiles | M: ≥ 3.41  F: ≥ 4.06  9.27–19.82 | M: ≤1.22  F: ≤1.19  0.08–2.95 | ng/ml  ng/ml | 0.67 (0.47–0.94)  1.52 (0.87-2.64)  1.15 (0.70–1.91) | 0.91 (0.81–1.02) | | Y  Y  Y | | Y | Y  Y  Y | Y  Y  Y | |  | Y |  |
| Insulin | Stolzenberg-Solomon R.Z et al.  Michaud DS et al. | 169  120 | Quartiles  Quartiles | >6.10  31.8 | <2.75  1.0 | μIU/Ml  μIU/Ml | 2.01 (1.03-3.93)  1.08 (0.57-2.04) |  | | Y  Y | |  | Y  Y | Y  Y | |  |  |  |
| Insulin resistance (HOMA-IR) | Stolzenberg-Solomon R.Z et al. |  | Quartiles | >1.53 | <0.70 |  | 1.69 (0.92-3.14) |  | | Y | |  | Y | Y | |  |  |  |
| Proinsulin | Wolpin BM et al. | 449 | Quintiles | 22.0 | 3.69 | pM | 2.22 (1.50 to 3.29) |  | | Y | | Y | Y | Y | |  |  |  |
| Proinsulin to insulin ratio | Wolpin BM et al. | 449 | Quintiles | 0.72 | 0.12 | pM / pM | 1.03 (0.70 to 1.51) |  | | Y | | Y | Y | Y | |  |  |  |

**Supplementary Table No. 3: Studies assessing glucose-related biomarkers and their association with PDAC risk**
