# Supplementary material for: The Role of Circulating Protein and Metabolite Biomarkers in the Development of Pancreatic Ductal Adenocarcinoma (PDAC): A Systematic Review and Meta-analysis
Source: Cancer Epidemiol Biomarkers Prev. 2021 Nov 22;31(5):1090–102. doi: 10.1158/1055-9965.EPI-21-0616 (PMC9377754; doi:10.1158/1055-9965.EPI-21-0616)
Supplement: Supplementary Data [file epi-21-0616_supp4.docx]

|  |  | Total cases n | Categorical | | | | | | Continuous | | Adjusted for/stratified by: | | | | | | | |  | | |
| --- | --- | --- | --- | --- | --- | --- | --- | --- | --- | --- | --- | --- | --- | --- | --- | --- | --- | --- | --- | --- | --- |
| Biomarkers |  |  | Categories | High | Reference | Units | RR/HR/OR (95% CI) | RR/HR (95% CI) | | Age | | Sex | BMI/WHR | Smoking | | Alcohol | | Diabetes | | |  |
| Vitamin C | Banim, PJR et al  Jeurnink SM. et al | 76  466 | Quartiles  Quartiles | > 242  >50.71 | > 41.0  <22.55 | μmol/l  μmol/l | 0.42 (0.20 to 0.91)  0.91 (0.55–1.51) |  | | Y  Y | | Y  Y |  | Y  Y | |  | | Y  Y | | |  |
| Serum B6 vitamers  Pyridoxal 5′-phosphate(PLP)  Pyridoxal (PL)  4-pyridoxic acid (PA)  PA/(PL + PLP) ratio (PAr) | Huang J.Y et al.  Chuang S.C et al  Schernhammer E et al.  Stolzenberg-Solomon RZ et al.  Huang J.Y et al.  Huang J.Y et al.  Huang J.Y et al. | 187  463  208  126  187  187  187 | Tertiles  Quintiles  Quartiles  Tertiles  Quartiles  Quartiles  Quartiles | >52.4  >54.82  >39.46  >24.0  >20.4  >0.39 | <20.0  ≤23.75  ≤26.34  <11.8  <8.8  <0.21 | nmol/L  nmol/L  pmol/mL  nmol/L  nmol/L  nmol/L  Ratio | 0.46 (0.23–0.92)  0.7(0.4–1.1)  0.87 (0.55–1.37)  0.43 (0.24–0.77)  0.82 (0.46–1.44)  0.94 (0.49–1.84)  1.07 (0.63–1.81) |  | | Y | |  | Y  Y  Y  Y  Y  Y | Y  Y  Y  Y  Y  Y | | Y  Y  Y  Y  Y | | Y  Y  Y  Y  Y  Y | | |  |
| Kynurenine pathway  Tryptophan  Kynurenine  Anthranilic acid  Kynurenic acid  3 hydroxykynurenine  Xanthurenic acid  3-hydroxyanthranilic acid  KA:HK ratio  XA:HK ratio  HAA:HK ratio  KTR  Neopterin  Picolinic acid | Huang J.Y et al.  Stolzenberg-Solomon R.Z et al.  Huang J.Y et al.  Huang J.Y al.  Huang J.Y et al.  Huang J.Y et al.  Huang J.Y et al.  Huang J.Y et al.  Huang J.Y et al.  Huang J.Y et al.  Huang J.Y et al.  Huang J.Y et al.  Huang J.Y et al.  Shu, X et al. | 187  479  187  187  187  187  187  187  187  187  187  187  187  226 | Tertile  Quartiles  Tertile  Tertile  Tertile  Tertile  Tertile  Tertile  Tertile  Tertile  Tertile  Tertile  Tertile  Tertile | <69.7  <1.45  <16.4  <46.4  <39.5  <14.7  <33.2  <1.00  <0.326  <0.739  <1.93  <13.8 | >80.1  >1.72  >23.0  >63.5  >52.6  >21.3  >45.0  >1.40  >0.451  >0.994  >2.28  >19.2 | µmol/L  µmol/L  nmol/L  nmol/L  nmol/L  nmol/L  nmol/L  ×100  nmol/L | 0.88 (0.56-1.39)  1.64 (1.10,2.46)  0.71 (0.42-1.19)  1.53 (0.88-2.67)  0.83 (0.51-1.34)  0.69 (0.42-1.12)  0.83 (0.53-1.32)  0.62 (0.39-1.00)  0.94 (0.57-1.53)  0.87 (0.54-1.39)  0.60 (0.37-0.98)  0.90 (0.53-1.52)  0.84 (0.51-1.4)  2.13 (0.88‐5.12) | 1.24 (1.08 to 1.42)  2.53 (1.61–3.95) | | Y  Y | | Y | Y  Y  Y  Y  Y  Y  Y  Y  Y  Y  Y  Y | Y  Y  Y  Y  Y  Y  Y  Y  Y  Y  Y  Y  Y | | Y  Y  Y  Y  Y  Y  Y  Y  Y  Y  Y  Y  Y | | Y  Y  Y  Y  Y  Y  Y  Y  Y  Y  Y  Y  Y | | |  |
| Vitamin D-binding protein  25(OH)D  25(OH)D:DBP molar ratio | Piper M.R et al.  Weinstein SJ et al.  Stolzenberg-Solomon RZ et al.  Stolzenberg-Solomon RZ et al.  Wolpin, BM et al.  Weinstein SJ et al. | 295  234  184  200  451  234 | Quintiles  Quartiles  Quintiles  Quintiles  Quintiles  Quartiles | ≥7149.4  >6721  >82.3    >65.5  > 81.05  >11.03 | <3670.4  ≤4026  ≤45.9  <32  < 45.64  ≤4.91 | nmol/L  nmol/L  nmol/L  nmol/L  nmol/L | 1.75 (0.91, 3.37)  0.70 (0.40–1.22)  1.45 (0.66–3.15)  2.92 (1.56- 5.48)  0.67 (0.46–0.97)  1.86 (0.97–3.56) |  | | Y  Y  Y  Y  Y | | Y  Y | Y  Y  Y  Y | | Y  Y  Y  Y  Y  Y | |  | | | Y  Y  Y  Y |  |
| Vitamin B12 | Arendt J. et al.  Schernhammer E et al.  Stolzenberg-Solomon RZ et al. | 844  208  126 | Quartiles  Tertiles | >1,000  <550 | 150–600  ≤427 | pmol/L  pg/Ml  pg/mL | 7.42 (4.51,12.21)  0.93 (0.58–1.49)  1.08 (0.64–1.81) |  | | Y  Y | | Y | Y  Y | Y  Y | | Y | | Y | | |  |
| Carotenoids  α-Carotene  β-Carotene  Lycopene  B-cryptoxanthin  Zeaxthantin  Lutein  Sum of carotenoids | Jeurnink SM. et al  Jeurnink SM. et al  Jeurnink SM. et al  Jeurnink SM. et al  Jeurnink SM. et al  Jeurnink SM. et al  Jeurnink SM. et al | 466  466  466  466  466  466  466 | Quartiles  Quartiles  Quartiles  Quartiles  Quartiles  Quartiles  Quartiles | >147.16  >678.86  >396.50  >495.52  >97.50  >390.84  >2174.47 | <55.67  <252.35  <163.14  <150.17  <46.24  <183.06  <1089.69 | nmol/L  nmol/L  nmol/L  nmol/L  nmol/L  nmol/L  nmol/L | 1.14 (0.71–1.85)  0.52 (0.31–0.88)  1.00 (0.62–1.62)  0.66 (0.39–1.13)  0.53 (0.30–0.94)  0.90 (0.54–1.50)  0.61 (0.36–1.04) |  | | Y  Y  Y  Y  Y  Y  Y | | Y  Y  Y  Y  Y  Y  Y |  | Y  Y  Y  Y  Y  Y  Y | |  | | Y  Y  Y  Y  Y  Y  Y | | |  |
| Retinol | Jeurnink SM. et al | 466 | Quartiles | >2.49 | <1.64 | μmol/l | 0.84 (0.50–1.40) |  | | Y | | Y |  | Y | |  | | Y | | |  |
| α-tocopherol | Jeurnink SM. et al  Stolzenberg-Solomon RZ et al. | 466  306 | Quartiles  Quintiles | <23.32  ≥14.2 | <23.32  <9.3 | μmol/l  mg/L | 0.62 (0.39–0.99)  0.52 (0.34, 0.80) | 0.91 (0.84, 0.99) | | Y  Y | | Y |  | Y  Y | |  | | Y  Y | | |  |
| γ-tocopherol | Jeurnink SM. et al | 466 | Quartiles | <1.86 | <1.86 | μmol/l | 0.92 (0.55–1.54) |  | | Y | | Y |  | Y | |  | | Y | | |  |

**Supplementary Table No. 4: Studies assessing nutrition-related biomarkers and their association with PDAC risk**
