# Supplementary material for: The Role of Circulating Protein and Metabolite Biomarkers in the Development of Pancreatic Ductal Adenocarcinoma (PDAC): A Systematic Review and Meta-analysis
Source: Cancer Epidemiol Biomarkers Prev. 2021 Nov 22;31(5):1090–102. doi: 10.1158/1055-9965.EPI-21-0616 (PMC9377754; doi:10.1158/1055-9965.EPI-21-0616)
Supplement: Supplementary Data [file epi-21-0616_supp5.docx]

|  |  | Total cases n | Categorical | | | | | Continuous | Adjusted for/stratified by: | | | | | |
| --- | --- | --- | --- | --- | --- | --- | --- | --- | --- | --- | --- | --- | --- | --- |
| Biomarkers |  |  | **Categories** | **High** | **Reference** | **Units** | **RR/HR/OR (95% CI)** | **RR/HR (95% CI)** | **Age** | **Sex** | **BMI/WHR** | **Smoking** | **Alcohol** | **Diabetes** |
| Cholesterol | Johansen, Dorthe et al.  Kitahara et al.  Ahn J et al.  Chen W.C et al.  Kabat G.C. et al | 862  2,575  273  1241  156 | Quintiles  Quartiles  Quintiles  Quartiles  Quartiles | M:7.6  F:7.6  ≥ 240  ≥276.7  >7 | M:4.5  F:4.4  < 160  <203.9  <4 | mmol/L  mmol/L  mg/dL  mg/dL  mmol/L  mg/dL | 0.70(0.53-0.93)  1.11(0.75-1.64)  M: 0.88 (0.74-1.05)  F: 0.96 (0.74-1.24)  0.96(0.67, 1.38)  0.68 (0.44-1.06)  0.76 (0.47-1.21) | 0.92 (0.84-1.00)  1.10 (0.99-1.2)  0.92 (0.85-1.00) | Y  Y  Y  Y  Y |  | Y  Y  Y  Y  Y  Y | Y  Y  Y  Y  Y  Y | Y  Y  Y  Y | Y |
| *HDL Cholesterol* | Ahn J et al.  Kabat G.C. et al.  Meinhold CL et al. | 273  156  305 | Quintiles  Quartiles  Quintiles | ≥55.3  ≥1.43 | <36.2  <0.93 | mg/dl  mg/Dl  nmol/L | 0.94(0.64, 1.40)  0.53 (0.31-0.89)  1.00 (0.70, 1.43) | 1.00 (0.96, 1.05) | Y  Y  Y |  | Y  Y  Y | Y  Y  Y | Y  Y |  |
| *LDL Cholesterol* | Kabat G.C. et al | 156 | Quartiles |  |  | mg/dL | 0.92 (0.58-1.46) |  | Y |  | Y | Y | Y |  |
| *Triglycerides* | Johansen, Dorthe et al.  Kabat G.C. et al | 862  156 | Quintiles  Quartiles | M:3.4  F:2.5 | M:0.8  F:0.6 | mmol/L  mmol/L  mg/dL | 1.13(0.84-1.52)  1.09(0.75-1.59)  1.19 (0.72-1.98) | 1.05 (0.96-1.1)  1.16 (1.04-1.9) | Y  Y  Y |  | Y  Y  Y | Y  Y  Y | Y |  |
| *Phospholipid fatty acids*  *Saturated fatty acids*  15:0 (pentadecanoic acid)  16:0 (palmitic acid)  17:0 (heptadecanoic acid)  18:0 (stearic acid)  *Cis monounsaturated fatty*  *acids*  16:1n-7 (palmitoleic acid)  18:1n-9 (oleic acid)  *Trans monounsaturated fatty*  *acids*  1n-9 t/12 t (elaidic acid)  18:1n-7 t (vaccenic acid)  *Cis n-6 polyunsaturated fatty*  *acids*  18:2n-6 (linoleic acid)  18:3n-6 (γ-linolenic acid)  20:3n-6 (di-homo-γ-linolenic acid  20:4n-6 (arachidonic acid)  *Conjugated linoleic acids*  9c11t + 10t12c  *Cis n-3 polyunsaturated fatty*  *acids*  18:3n-3 (α-linolenic acid)  20:5n-3 (eicosapentaenoic acid) 22:5n-3 (docosapentaenoic acid)  22:6n-3 (docosahexaenoic acid) Total saturated fatty acids  Total cis monounsaturated fatty acids  Total cis n-6 polyunsaturated fatty acids  Total cis n-3 polyunsaturated fatty acids  Long-chain n-6/n-3 PUFA ratio  Total trans ruminant fatty acids  Total trans industrial fatty acid  *Desaturation index*  DI_16_ (16:1n-7c/16:0)  DI_18_ (18:1n-9c/18:0) | Matejcic, M et al.  Matejcic, M et al.  Matejcic, M et al.  Matejcic, M et al.  Matejcic, M et al.  Matejcic, M et al.  Matejcic, M et al.  Matejcic, M et al.  Matejcic, M et al.  Matejcic, M et al.  Matejcic, M et al.  Matejcic, M et al.  Matejcic, M et al.  Matejcic, M et al.  Matejcic, M et al.  Matejcic, M et al.  Matejcic, M et al.  Matejcic, M et al.  Matejcic, M et al.  Matejcic, M et al.  Matejcic, M et al.  Matejcic, M et al.  Matejcic, M et al.  Matejcic, M et al.  Matejcic, M et al.  Matejcic, M et al | 375  375  375  375  375  375  375  375  375  375  375  375  375  375  375  375  375  375  375  375  375  375  375  375  375  375 | Tertiles  Tertiles  Tertiles  Tertiles  Tertiles  Tertiles  Tertiles  Tertiles  Tertiles  Tertiles  Tertiles  Tertiles  Tertiles  Tertiles  Tertiles  Tertiles  Tertiles  Tertiles  Tertiles  Tertiles  Tertiles  Tertiles  Tertiles  Tertiles  Tertiles  Tertiles |  |  |  | 0.63 (0.38; 1.04)  1.05 (0.69; 1.59)  0.63 (0.41; 0.98)  0.92 (0.61; 1.38)  1.17 (0.77; 1.79)  1.10 (0.71; 1.69)  1.24 (0.72; 2.15)  1.12 (0.66; 1.89)  0.93 (0.61; 1.43)  1.10 (0.71; 1.69)  1.14 (0.75; 1.73)  1.14 (0.76; 1.71)  0.70 (0.42; 1.16)  0.60 (0.39; 0.92)  0.71 (0.48; 1.07)  0.52 (0.32; 0.85)  1.14 (0.76; 1.72)  0.99 (0.62; 1.59)  1.07 (0.70; 1.64)  0.87 (0.58; 1.30)  0.92 (0.62; 1.35)  1.02 (0.68; 1.54)  0.84 (0.50; 1.42)  1.07 (0.62; 1.84)  1.09 (0.70; 1.70)  1.18 (0.78; 1.79) | 0.72 (0.42; 1.26)  0.32 (0.13; 0.82)  0.50 (0.06; 3.85)  1.70 (1.00; 2.87)  1.30 (0.52; 3.24)  1.11 (0.76; 1.62)  1.05 (0.74; 1.49)  1.15 (0.68; 1.93)  1.13 (0.79; 1.60)  1.38 (0.67; 2.86)  0.89 (0.38; 2.10)  0.87 (0.58; 1.32)  0.59 (0.38; 0.92)  0.95 (0.69; 1.30)  0.43 (0.18; 1.04)  1.10 (0.62; 1.94)  1.49 (0.55; 4.09)  0.76 (0.11; 5.54)  0.95 (0.50; 1.80)  1.03 (0.61; 1.73)  1.08 (0.59; 1.97)  1.15 (0.71; 1.87)  1.91 (1.07; 3.39)  1.31 (0.62; 2.74) |  |  | Y  Y  Y  Y  Y  Y  Y  Y  Y  Y  Y  Y  Y  Y  Y  Y  Y  Y  Y  Y  Y  Y  Y  Y  Y  Y | Y  Y  Y  Y  Y  Y  Y  Y  Y  Y  Y  Y  Y  Y  Y  Y  Y  Y  Y  Y  Y  Y  Y  Y  Y  Y | Y  Y  Y  Y  Y  Y  Y  Y  Y  Y  Y  Y  Y  Y  Y  Y  Y  Y  Y  Y  Y  Y  Y  Y  Y  Y | Y  Y  Y  Y  Y  Y  Y  Y  Y  Y  Y  Y  Y  Y  Y  Y  Y  Y  Y  Y  Y  Y  Y  Y  Y  Y |
| *Lipids*  Tetracosanoic acid  PC(18:1/18:4)  Coumarin  PC(p-18:0/22:6)  PE(22:6/16:0)  PS(18:0/18:0)  PC(15:0/18:2)  PC(22:5/14:0)  PE(22:6/p-18:1)  Sphingosine  7-α-Hydroxy-3-oxo-4-cholestenoate | Shu, X et al.  Shu, X et al.  Shu, X et al.  Shu, X et al.  Shu, X et al.  Shu, X et al.  Shu, X et al.  Shu, X et al.  Shu, X et al.  Stolzenberg-Solomon R.Z et al.  Stolzenberg-Solomon R.Z et al. | 226  226  226  226  226  226  226  226  226  479  479 | Tertiles  Tertiles  Tertiles  Tertiles  Tertiles  Tertiles  Tertiles  Tertiles  Tertiles  Quartiles  Quartiles |  |  |  | 0.36 (0.20‐0.64)  0.47 (0.25‐0.87)  2.45 (1.34‐4.49)  0.54 (0.31‐0.96)  0.27 (0.13‐0.53)  0.39 (0.19‐0.77)  1.78 (0.83‐3.81)  0.31 (0.16‐0.60)  0.18 (0.08‐0.40) | 0.48 (0.36–0.64)  0.50 (0.38–0.66)  1.96 (1.47–2.61)  0.53 (0.40–0.71)  0.49 (0.35–0.69)  0.44 (0.30–0.66)  2.32 (1.49–3.60)  0.65 (0.51–0.82)  0.58 (0.42–0.79)  1.34 (1.13 to 1.59)  1.22 (1.07 to 1.40) | Y  Y  Y  Y  Y  Y  Y  Y  Y  Y  Y | Y  Y |  | Y  Y  Y  Y  Y  Y  Y  Y  Y | Y  Y  Y  Y  Y  Y  Y  Y  Y | Y  Y  Y  Y  Y  Y  Y  Y  Y |
| Branched chain amino acids  Valine  ( ≥10 years)  Valine  Leucine (≥10 years)  Leucine  Isoleucine ( ≥10 years)  Isoleucine  BCAAs | Shu, X et al.  Katagiri. et al  Mayers et al.  Shu, X et al.  Katagiri.et al  Mayers et al.  Shu, X et al.  Katagiri. et al  Mayers et al.  Katagiri. et al  Mayers et al. | 226  170  453  226  170  453  226  170  453  170  453 | Quartiles  Quintiles  Quartiles  Quintiles  Quartiles  Quintiles  Quartiles  Quintiles | ≥ 221.4  ≥ 125.2  ≥ 70.8  ≥ 411.3 | ≤ 163.7  ≤ 89.4  ≤ 44.1  ≤ 303.2 | µmol/L  µmol/L  µmol/L  µmol/L | 2.89 (1.43–5.84)  1.90 (1.28–2.81)  2.14 (1.10–4.15)  1.97 (1.29–2.99)  2.07 (0.99–4.31)  2.00 (1.31–3.05)  2.43 (1.21–4.90)  2.01 (1.34–3.03) | 0.81 (0.45‐1.45)  1.27 (1.01–1.61)  1.20 (1.06–1.37)  0.84 (0.48‐1.48)  1.31 (1.05–1.63)  1.28 (1.11–1.48)  0.91 (0.51‐1.61)  1.38 (1.09–1.76)  1.28 (1.13–1.46)  1.32 (1.05–1.67)  1.27 (1.11–1.46) | Y  Y  Y  Y  Y  Y  Y  Y  Y  Y  Y |  | Y  Y  Y  Y  Y  Y  Y  Y | Y  Y  Y  Y  Y  Y  Y | Y  Y  Y  Y  Y  Y  Y | Y  Y  Y  Y  Y  Y  Y  Y  Y  Y  Y |
| One carbon metabolism  Total homocysteine  Folate  Methionine  Cobalamin  Riboflavin  Flavin mononucleotide  Dimethylglycine (DMG)  Trimethylamine-N-oxide (TMAO)  Choline  Betaine  Total methyl donors | Chuang S.C et al  Schernhammer E et al.  Stolzenberg-Solomon R.Z et al.  Chuang S.C et al  Schernhammer E et al.  Stolzenberg-Solomon R.Z et al.  Chuang S.C et al  Huang J. et al  Chuang S.C et al  Chuang S.C et al  Chuang S.C et al  Huang J. et al  Huang J. et al  Huang J. et al  Huang J. et al  Huang J. et al | 463  208  126  463  208  126  463  187  463  463  463  187  187  187  187  187 | Quintiles  Quartiles  Tertiles  Quintiles  Quartiles  Tertiles  Quintiles  Quartiles  Quintiles  Quintiles  Quintiles  Quartiles  Quartiles  Quartiles  Quartiles  Quartiles | >12.33  >12.51  >20.17  >4.45  >30.24  >493.61  >29.22  >11.55 | ≤7.57  ≤9.99  ≤9.02  ≤3.33  ≤20.67  ≤267.31  ≤10.24  ≤4.65 | μmol/L  μmol/L  μmol/L  nmol/L  ng/mL  ng/mL  μmol/L  nmol/l  pmol/L  nmol/L  nmol/L  nmol/l  nmol/l  nmol/l  nmol/l  nmol/l | 0.7(0.7–1.5)  1.37 (0.85–2.20)  0.87 (0.49–1.53)  0.8(0.5–1.4)  1.22 (0.77–1.95)  0.53 (0.30–0.93)  1.0(0.7–1.4)  0.40 (0.23–0.70)  0.9(0.6–1.5)  0.9(0.5–1.4)  0.7(0.4–1.3)  0.93 (0.54–1.60)  2.36 (1.30–4.26)  0.42 (0.20–0.85)  0.59 (0.35–0.98)  0.38 (0.21–0.68) | 0.96 (0.94–0.99)  0.99 (0.92–1.07)  1.01 (0.99–1.04)  0.42 (0.20–0.85)  0.59 (0.35–0.98)  0.99 (0.98–1.00) | Y  Y |  | Y  Y  Y  Y  Y  Y  Y  Y  Y  Y  Y  Y  Y  Y | Y  Y  Y  Y  Y  Y  Y  Y  Y  Y  Y  Y  Y  Y | Y  Y  Y  Y  Y  Y  Y  Y  Y  Y  Y  Y | Y  Y  Y  Y  Y  Y  Y  Y  Y  Y  Y  Y  Y  Y |
| Aspartate aminotransferase  Alanine aminotransferase | De Gonzalez A.B et al  De Gonzalez A.B et al | 2194  2194 |  | 40+  40+ | <20  <20 |  | 1.33 (1.14-1.55)  1.34 (1.16-1.56) |  | Y  Y | Y  Y | Y  Y | Y  Y |  |  |
| Dipeptide  Glycylvaline  Aspartylphenylalanine  Tyrosylglutamine  Pyroglutamylglycine  α-Glutamyltyrosine  Phenylalanylphenylalanine  Phenylalanylleucine  Tryptophylglutamate | Stolzenberg-Solomon, R Z et al.  Stolzenberg-Solomon, R Z et al  Stolzenberg-Solomon, R Z et al  Stolzenberg-Solomon, R Z et al  Stolzenberg-Solomon, R Z et  al  Stolzenberg-Solomon, R Z et al  Stolzenberg-Solomon, R Z et al  Stolzenberg-Solomon, R Z et al | 479  479  479  479  479  479  479  479 | Quartiles  Quartiles  Quartiles  Quartiles  Quartiles  Quartiles  Quartiles  Quartiles |  |  |  | 2.95 (1.96,4.45)  2.17 (1.47,3.2)  0.49 (0.33,0.73)  2.20 (1.49,3.24)  0.46 (0.31,0.68)  2.26 (1.49,3.43)  2.27 (1.50,3.43)  1.79 (1.22,2.63) | 1.46 (1.28 to 1.67)  1.38 (1.21 to 1.59)  0.72 (0.63 to 0.83)  1.35 (1.18 to 1.53)  0.74 (0.65 to 0.84)  1.33 (1.17 to 1.52)  1.33 (1.16 to 1.53)  1.30 (1.15 to 1.48) | Y  Y  Y  Y  Y  Y  Y  Y | Y  Y  Y  Y  Y  Y  Y  Y |  |  |  |  |
| Purine metabolism, guanine containing  7-Methylguanine  Guanine  N2,N2-dimethylguanosine | Stolzenberg-Solomon, R Z et al  Stolzenberg-Solomon, R Z et al  Stolzenberg-Solomon, R Z et al | 479  479  479 | Quartiles  Quartiles  Quartiles |  |  |  | 1.92 (1.26,2.93)  0.43 (0.23,0.84)  2.20 (1.37,3.53) | 1.27 (1.10 to 1.47)  0.74 (0.61 to 0.91)  1.24 (1.07 to 1.44) | Y  Y  Y | Y  Y  Y |  |  |  |  |
| Alanine and aspartate metabolism  Aspartate  3-Ureidopropionate | Stolzenberg-Solomon, R Z et al  Stolzenberg-Solomon, R Z et al | 479  479 | Quartiles  Quartiles |  |  |  | 1.96 (1.35,2.85)  1.69 (1.14,2.51) | 1.31 (1.15 to 1.49)  1.26 (1.10 to 1.44) | Y  Y | Y  Y |  |  |  |  |
| γ-Glutamyl amino acid  γ-Glutamylglutamate  γ-Glutamylisoleucine  γ-Glutamylphenylalanine | Stolzenberg-Solomon, R Z et al  Stolzenberg-Solomon, R Z et al  Stolzenberg-Solomon, R Z et al | 479  479  479 | Quartiles  Quartiles  Quartiles |  |  |  | 1.78 (1.18,2.69)  1.83 (1.21,2.78)  2.16 (1.41,3.30) | 1.27 (1.11 to 1.46)  1.25 (1.09 to 1.43)  1.26 (1.10 to 1.45) | Y  Y  Y | Y  Y  Y |  |  |  |  |
| Phenylalanine and tyrosine metabolism  Phenylalanine  O-cresol sulfate  3-Methoxytyrosine | Stolzenberg-Solomon, R Z et al  Stolzenberg-Solomon, R Z et al  Stolzenberg-Solomon, R Z et al | 479  479  479 | Quartiles  Quartiles  Quartiles |  |  |  | 1.72 (1.13,2.63)  1.28 (0.86,1.91)  2.23 (1.45,3.44) | 1.26 (1.10 to 1.45)  1.24 (1.09 to 1.42)  1.26 (1.09 to 1.46) | Y  Y  Y | Y  Y  Y |  |  |  |  |
| Fibrinogen cleavage peptide  DSGEGDFXAEGGGVR | Stolzenberg-Solomon, R Z et al | 479 | Quartiles |  |  |  | 0.51 (0.35,0.76) | 0.74 (0.65 to 0.85) | Y | Y |  |  |  |  |
| Glutathione metabolism  Cysteine-glutathione  disulfide | Stolzenberg-Solomon, R Z et al | 479 | Quartiles |  |  |  | 0.47 (0.31,0.69) | 0.75 (0.65 to 0.85) | Y | Y |  |  |  |  |
| Glutamate metabolism  Glutamate | Stolzenberg-Solomon, R Z et al | 479 | Quartiles |  |  |  | 2.46 (1.59,3.80) | 1.31 (1.14 to 1.49) | Y | Y |  |  |  |  |
| Sugar metabolism  Mannose | Stolzenberg-Solomon, R Z et al | 479 | Quartiles |  |  |  | 1.96 (1.26,3.04) | 1.37 (1.16 to 1.62) | Y | Y |  |  |  |  |
| Trptophan metabolism  C-glycosyltryptophan | Stolzenberg-Solomon R.Z et al. | 479 | Quartiles |  |  |  | 1.87 (1.22,2.89) | 1.26 (1.09 to 1.46) | Y | Y |  |  |  |  |

**Supplementary Table No. 5: Studies assessing metabolism-related biomarkers and their association with PDAC risk**
