# Supplementary material for: The Role of Circulating Protein and Metabolite Biomarkers in the Development of Pancreatic Ductal Adenocarcinoma (PDAC): A Systematic Review and Meta-analysis
Source: Cancer Epidemiol Biomarkers Prev. 2021 Nov 22;31(5):1090–102. doi: 10.1158/1055-9965.EPI-21-0616 (PMC9377754; doi:10.1158/1055-9965.EPI-21-0616)
Supplement: Supplementary Data [file epi-21-0616_supp6.docx]

|  |  | Total cases n | Categorical | | | | | Continuous | Adjusted for/stratified by: | | | | |  |
| --- | --- | --- | --- | --- | --- | --- | --- | --- | --- | --- | --- | --- | --- | --- |
| Biomarkers | **Reference** |  | Categories | High | Reference | Units | RR/HR/OR (95% CI) | RR/HR (95% CI) | Age | Sex | BMI/WHR | Smoking | Alcohol | Diabetes |
| Prostaglandin E2 metabolites (PGE-M) | Cui Y et al. | 239 | Quartile | NA | NA | NA | 1.94 (1.07–3.51) |  |  |  | Y | Y | Y |  |
| C-Reactive Protein | Douglas, JB et al.  Bao Y. et al  Sollie S et al.  Grote V.A et al. | 493  468  286  455 | Quintiles  Quintiles  Quartiles | >12.59  ≥4.3  ≥ 10  2.06–34.07 | ≤1.67  <0.5  <10  0.02–0.51 | ng/ml  mg/ml  mg/L  mg l^-1^ | 1.00 (0.69–1.46)  1.10 (0.74-1.63)  1.32 (1.00–1.74)  1.02 (0.66–1.57) | 0.98 (0.95–1.01)  0.99 (0.98-1.01) | Y  Y  Y | Y  Y  Y  Y | Y  Y | Y  Y  Y |  | Y |
| Advanced glycation end products (AGE):  Nᵋ-(carboxymethyl) lysine (CML)  Endogenous secreted receptor for AGE (esRAGE)  CML/esRAGE  sRAGE  CML/sRAGE | Grote V.A et al.  Jiao, Li et al.  Grote V.A et al.  Grote V.A et al  Jiao, Li et al.  White, DL et al.  Jiao, Li et al. | 454  255  454  454  255  472  255 | Quartiles  Quintiles  Quartiles  Quartiles  Quintiles  Quartiles  Quintiles | 1,018–1,819  ≥693  672–1,793  66–7.55  ≥794  1737–6999  ≥1399 | 328–55  <437  109–30  0.34–1.0  <374  242–1020  <672 | ng/L  ng/ml  pg/mL  Ratio  ng/ml  pg/mL  Ratio | 0.77 (0.42–1.4)  0.45 (0.27-0.73)  1.09 (0.67–1.78)  0.80 (0.48–1.3)  0.40 (0.24-0.67)  0.74 (0.52–1.05)  2.02 (1.20-3.38) | 0.85 (0.57–1.28)  1.04 (0.83–1.30)  0.94 (0.75–1.17) | Y  Y  Y |  | Y  Y  Y  Y  Y  Y  Y | Y  Y  Y  Y  Y  Y  Y |  | Y |
| Transforming growth factor-β1 (TGF-β1) | Jacobs, Eric J et al. | 729 | Quartiles |  |  | ng/ml | 1.36 (0.98-1.88) | 1.07 (0.97-1.19) | Y |  | Y | Y |  | Y |
| Adiponectin  HMW adiponectin | Nogueira L et al  Grote V.A et al  Bao Y. et al.  Nogueira L et al. | 758  452  468  758 | Quintiles  Quartiles  Quintiles  Quintiles | M: ≥ 10,634  F: ≥ 19,579  M: 10.16–20.16  F: 14.51–29.14  ≥10.9  M: ≤ 6,439  F: ≤ 18,465 | M: ≤ 4,991  F: ≤ 8,781  M: 2.06–6.46  F: 0.07–8.02  < 4.4  M: ≤ 1,838  F: ≤ 4,165 | ng/ml  µg/ml  µg/ml  µg/ml  ng/ml | 1.07 (0.77–1.48)  1.10 (0.69–1.75)  0.63 (0.43-0.92)  1.60 (1.00–2.56) | 1.00 (0.90–1.11)  1.02 (0.98–1.06)  1.09 (0.94–1.27) | Y  Y |  | Y  Y  Y  Y | Y  Y  Y  Y |  | Y  Y  Y |
| Leptin | Stolzenberg-Solomon R et al.  White, DL et al  Babic A et al. | 731  472  470 | Quintiles  Quartiles  Quintiles | M: ≥11.67  F: ≥40.78  39.7–36.5  M: ≥11.8  F: ≥37.8 | M: <2.89  F: <10.82  0.7–13.3  M: ≤3.0  F: ≤9.5 | ng/ml  ng/ml  ng/ml  ng/ml  ng/ml | 1.13 (0.75, 1.71)  0.77 (0.50–1.20)  2.54 (1.13–5.72)  0.94 (0.55–1.62) | 1.06 (0.93, 1.22) | Y  Y  Y |  | Y  Y  Y  Y | Y  Y  Y  Y |  | Y  Y  Y  Y |
| Adiponectin/Leptin | White, DL et al | 472 | Quartiles | 1050–2800 | 208–227 | Ratio | 0.90 (0.57–1.42) |  |  |  | Y | Y |  | Y |
| MCP1 | White, DL et al | 472 | Quartiles | 279–3043 | 43.3–165 | pg/mL | 1.16 (0.81–1.65) |  |  |  | Y | Y |  | Y |
| PAI1 | White, DL et al | 472 | Quartiles | 93.7–205.7 | 3.0–59.4 | ng/ml | 1.24 (0.86–1.80) |  |  |  | Y | Y |  | Y |
| Interleukin-6 | Babic A et al.  Grote V.A et al | 470  455 | Quintiles  Quartiles | ≥2.2  0.16–0.94 | <0.7  2.66–9.66 | pg/mL  pg l^-1^ | 1.19 (0.81-1.76)  1.01 (0.64–1.61) | 1.01 (0.99-1.04) | Y | Y  Y | Y  Y | Y  Y |  | Y |
| Tumor necrosis factor-  α-receptor I | Grote V.A et al | 455 | Quartiles | 1.59–2.95 | 0.75-1.13 | ng l^-1^ | 0.95 (0.58–1.55) |  | Y | Y | Y | Y |  |  |
| Tumor necrosis factor-  α-receptor II | Babic A et al.  Grote V.A et al | 470  455 | Quintiles  Quartiles | ≥3.5  0.83–1.95 | <2.1  2.69–4.82 | mg/mL  ng l^-1^ | 0.88 (0.58-1.33)  1.42 (0.89–2.27) | 0.92 (0.82-1.04) | Y | Y  Y | Y  Y | Y  Y |  | Y  Y |
| Albumin | Sollie S et al. | 286 |  | <40 | ≥ 40 | g/L | 1.11 (0.82–1.50) |  | Y | Y |  |  |  |  |
| Haptoglobin | Sollie S et al. | 286 |  | ≥ 1.4 | <1.4 | g/L | 2.23 (1.72–2.88) |  | Y | Y |  |  |  |  |

**Supplementary Table No. 6: Studies assessing inflammation-related biomarkers and their association with PDAC risk**
