# Supplementary material for: The Role of Circulating Protein and Metabolite Biomarkers in the Development of Pancreatic Ductal Adenocarcinoma (PDAC): A Systematic Review and Meta-analysis
Source: Cancer Epidemiol Biomarkers Prev. 2021 Nov 22;31(5):1090–102. doi: 10.1158/1055-9965.EPI-21-0616 (PMC9377754; doi:10.1158/1055-9965.EPI-21-0616)
Supplement: Supplementary Data [file epi-21-0616_supp7.docx]

|  |  | Total cases n | Categorical | | | | | Continuous | Adjusted for/stratified by: | | | | | | |
| --- | --- | --- | --- | --- | --- | --- | --- | --- | --- | --- | --- | --- | --- | --- | --- |
| Biomarkers |  |  | **Categories** | **High** | **Reference** | **Units** | **RR/HR/OR (95% CI)** | **RR/HR (95% CI)** | **Age** | **Sex** | **BMI/WHR** | **Smoking** | **Alcohol** | **Diabetes** |  |
| Pepsinogen I (SPGI) | Laiyemo A.O et al. | 227 |  | < 25 (low) | ≥25 | μg/L | 1.04 (0.60 – 1.80) |  | Y |  | Y | Y |  | Y |  |
| Tobacco metabolite  Cotinine  Hydroxycotinine  Cotinine N-oxide | Leenders, Max et al.  Stolzenberg-Solomon, R Z et al  Stolzenberg-Solomon, R Z et al  Stolzenberg-Solomon, R Z et al | 146  479  479  479 | Quintiles  Quartiles  Quartiles  Quartiles | >1187.8 | <55 | nmol/L | 3.66(1.44-9.26)  1.49 (0.96,2.31)  2.00 (0.43,9.18)  1.91 (1.21,3.00) | 1.33(1.11-1.60)  1.26 (1.10 to 1.44)  1.23 (1.08 to 1.41)  1.22 (1.06 to 1.39) | Y  Y  Y | Y  Y  Y | Y |  | Y | Y |  |
| Autoantibodies to Ezrin | Sun, YQ et al. | 73 | Tertiles | >0.767 | <0.495 | O.D. values | 1.15 (0.57,2.33) |  | Y | Y |  |  |  | Y |  |
| Selenium | Chatterjee S et al. | 302 |  | >160 | ≤ 125 | nmol/L | 0.74 (0.46–1.20) | 0.66 (0.32–1.37) | Y | Y |  | Y |  | Y |  |
| Inorganic phosphate | Wulaningsih W et al. | 762 | Quartiles | M: ≥1.14  F: ≥1.19 | M: 0.92  F: 0.99 | nmol/L  nmol/L | 1.41 (1.05 – 1.88)  1.24 (0.92 – 1.68) | 1.11 (1.02 – 1.22)  1.04 (0.93 – 1.18) | Y  Y |  |  |  |  | Y  Y |  |
| Iron  Total iron binding capacity (TIBC) | Gaur A. et al.  Gaur A. et al. | 197  197 | Quartiles  Quartiles | ≥22  ≥67 | <14  <42 | µmol/L  µmol/L | 0.98 (0.63–1.52)  1.36 (0.91–2.06) | 1.03 (0.89–1.20)  1.12 (0.97–1.30) | Y  Y | Y  Y |  |  |  |  |  |
| Forms of trypsinogen  Anionic trypsinogen (HAT)  Cationic trypsinogen (HCT)  The sum of trypsinogens (HAT + HCT)  HAT/HCT  Pancreatic secretory trypsin inhibitor (PSTI)  (HAT + HCT)/PSTI | Johansen D et al  Johansen D et al  Johansen D et al  Johansen D et al  Johansen D et al  Johansen D et al | 84  84  84  84  84  84 |  |  |  | μg/L  μg/L  μg/L  Ratio  μg/L  Ratio |  | 1.06 (0.93–1.21)  0.98 (0.82–1.17)  1.02 (0.94–1.10)  1.05 (0.95–1.17)  0.98 (0.70–1.37)  1.01 (0.99–1.03) | Y  Y  Y  Y  Y  Y | Y  Y  Y  Y  Y  Y | Y  Y  Y  Y  Y  Y | Y  Y  Y  Y  Y  Y |  |  |  |
| Salicylurate | Khalaf N et al. | 396 | Quintiles |  |  |  | 1.08 (0.72, 1.61) |  |  |  | Y |  | Y | Y |  |
| Immunoglobulins  Immunoglobulin E  Immunoglobulin G  Immunoglobulin M  Immunoglobulin A | Olson S.H. et al  Sollie S et al  Sollie S et al  Sollie S et al | 283  689  689  689 |  | >310  ≥15.00  ≥1.40  ≥3.66 | <25  6.10–14.99  <1.40  <3.66 | kU/L  g/L  g/L  g/L | 1.42 (0.85–2.37)  0.82 (0.64–1.05)  0.82 (0.50–1.35)  0.70 (0.39–1.26) | 1.04 (0.95–1.15) | Y  Y  Y | Y  Y  Y |  | Y |  |  |  |
| gamma-Glutamyltransferase | Mok Y. et al  Tsuboya et al. | 67 | Quartiles | M: ≥40  F: ≥40  ≥31 | M: <12  F: <12  <13 | IU/L  IU/L  IU/L | 1.14 (0.95–1.36)  1.26 (0.94–1.69)  1.89 (0.81–4.38) | 1.05 (1.03–1.08)  1.06 (0.94–1.21) | Y  Y  Y | Y | Y  Y  Y | Y  Y  Y | Y  Y  Y | Y  Y |  |

**Supplementary Table No. 7: Studies assessing miscellaneous biomarkers and their association with PDAC risk**
