# Supplementary figures and images for: The Role of Circulating Protein and Metabolite Biomarkers in the Development of Pancreatic Ductal Adenocarcinoma (PDAC): A Systematic Review and Meta-analysis
Source: Cancer Epidemiol Biomarkers Prev. 2021 Nov 22;31(5):1090–102. doi: 10.1158/1055-9965.EPI-21-0616 (PMC9377754; doi:10.1158/1055-9965.EPI-21-0616)

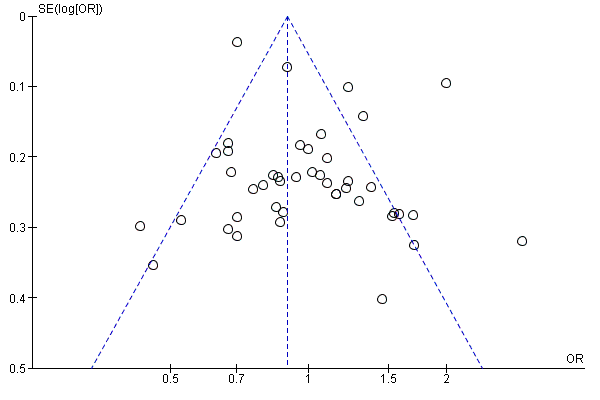


**Supplementary Figure 1: Funnel plot of all the studies included in the meta-analysis**

Supplement: Supplementary Data [file epi-21-0616_suppfigs1.docx]
